# TANDEM ZINC-FINGER/PLUS3 regulates phytochrome B abundance and signaling to fine-tune hypocotyl growth

**DOI:** 10.1093/plcell/koac236

**Published:** 2022-08-05

**Authors:** Weiwei Fang, Elisa Vellutini, Giorgio Perrella, Eirini Kaiserli

**Affiliations:** School of Molecular Biosciences, College of Medical, Veterinary and Life Sciences, University of Glasgow, Glasgow G12 8QQ, UK; School of Molecular Biosciences, College of Medical, Veterinary and Life Sciences, University of Glasgow, Glasgow G12 8QQ, UK; School of Molecular Biosciences, College of Medical, Veterinary and Life Sciences, University of Glasgow, Glasgow G12 8QQ, UK; School of Molecular Biosciences, College of Medical, Veterinary and Life Sciences, University of Glasgow, Glasgow G12 8QQ, UK

## Abstract

TANDEM ZINC-FINGER/PLUS3 (TZP) is a transcriptional regulator that acts at the crossroads of light and photoperiodic signaling. Here, we unveil a role for TZP in fine-tuning hypocotyl elongation under red light and long-day conditions. We provide genetic evidence for a synergistic action between TZP and PHOTOPERIODIC CONTROL OF HYPOCOTYL 1 (PCH1) in regulating the protein abundance of PHYTOCHROME INTERACTING FACTOR 4 (PIF4) and downstream gene expression in response to red light and long days (LDs). Furthermore, we show that TZP is a positive regulator of the red/far-red light receptor and thermosensor phytochrome B (phyB) by promoting phyB protein abundance, nuclear body formation, and signaling. Our data therefore assign a function to TZP in regulating two key red light signaling components, phyB and PIF4, but also uncover a new role for PCH1 in regulating hypocotyl elongation in LDs. Our findings provide a framework for the understanding of the mechanisms associated with the TZP signal integration network and their importance for optimizing plant growth and adaptation to a changing environment.

IN A NUTSHELL **Background:** Plants sense, respond, and adapt to the environment by changing their body shape and life cycle timing. Plant photoreceptors sense and communicate changes in light to proteins that control growth and life cycle events. Phytochrome B (phyB) detects red light and warmer temperatures and works with the transcription factor PHYTOCHROME INTERACTING FACTOR 4 (PIF4), the scaffold protein PHOTOPERIODIC CONTROL OF HYPOCOTYL1 (PCH1), and the transcriptional regulator TANDEM ZINC-FINGER/PLUS3 (TZP). These four proteins meet up in nuclear neighborhoods and help seedlings adapt to changes in light and temperature. TZP controls gene expression and seedling growth in blue and far-red light. Very little is known about the role of TZP in communicating with phyB, PIF4, and PCH1 to optimize seedling growth in red light and during day/night cycles.

**Question:** How does TZP influence phyB, PIF4, and PCH1 in regulating growth in response to red light? Is TZP an information hub for different light signals in Arabidopsis?

**Findings:** We reveal a new role for TZP in fine-tuning seedling growth under red light and long days (LDs) through a reciprocal relationship with PCH1. We also show that TZP affects the abundance and residency of phyB in nuclear neighborhoods and influences how much PIF4 protein is available in response to red light and LDs. The effect of TZP on phyB, PIF4, and PCH1 regulation leads to changes in seedling architecture and growth. Overall, we discovered that TZP as a positive regulator of red light signaling by enhancing the communication between TZP, phyB, and PCH1. Therefore, TZP is key in connecting information from different light qualities (blue, red, and far-red) and seasonal changes to optimize growth.

**Next steps:** Investigating how TZP regulates phyB protein independently or collaboratively with PCH1 will provide more information on their relationship and function. We are currently exploring whether TZP is a hub for light and temperature information and a potential candidate for improving plant survival to changing light availability and climate change.

## Introduction

Light is one of the most important environmental factors that fine-tunes plant growth and development. In addition to providing the main source of energy for photosynthesis, light also acts as a stimulus that triggers signaling cascades to optimize plant adaptation to fluctuating environmental regimes. Plant photoreceptors and downstream signaling components integrate information on changes in light quality, intensity, direction, and duration within a 24-h period (photoperiod), which is key for modulating major developmental transitions such as seed germination, de-etiolation, and flowering (Quail, 2002; Chory, 2010; Chen and Chory, 2011; Kaiserli and Chory, 2016).

Phytochromes (phyA–E) are red/far-red light receptors that control most aspects of development throughout the life cycle of a plant (Quail, 2002; Cheng et al., 2021). Phytochrome B (phyB) is the major red-light photoreceptor that also functions as a thermosensor in Arabidopsis (*Arabidopsis thaliana*) (Jung et al., 2016; Legris et al., 2016). An increasing number of protein components have been identified as important integrators of light responses acting downstream of phyB. Members from the PHYTOCHROME INTERACTING FACTOR (PIF) family of basic helix–loop–helix (bHLH) transcription factor (TF) family are negative regulators of photomorphogenesis and positive regulators of growth in response to shade avoidance (Leivar and Quail, 2011; Cheng et al., 2021). Photoactivated phytochromes initially translocate from the cytosol to the nucleus, associate with PIFs, and control PIF post-translational modifications, stability, and activity, which ultimately leads to the initiation of photomorphogenesis (Leivar et al., 2012; Park et al., 2018; Cheng et al., 2021). In particular, PIF4 (PIF4) plays a major role in the crosstalk between light, temperature, and phytohormone signal transduction pathways (Di et al., 2016; Casal and Questa, 2018; Hayes et al., 2021). In addition to TFs, phytochromes associate with nuclear proteins involved in light and photoperiodic signaling such as PHOTOPERIODIC CONTROL OF HYPOCOTYL1 (PCH1), EARLY FLOWERING3 (ELF3), and TANDEM ZINC-FINGER/PLUS3 (TZP) (Huang et al., 2016b).

TZP was originally identified as a morning-specific growth-promoting factor through quantitative trait locus (QTL) mapping between the Bay-0 and Shahdara Arabidopsis accessions (Loudet et al., 2008). Subsequent studies reported that TZP functions as a transcriptional regulator controlling hypocotyl elongation and flowering initiation (Kaiserli et al., 2015; Perrella et al., 2018). In vitro and in planta studies have reported that TZP associates with components of the evening complex (EC) and directly interacts with phytochromes (Kaiserli et al., 2015; Huang et al., 2016a; Zhang et al., 2018). More specifically, phyB is essential for the recruitment of TZP to subnuclear foci (also referred to as nuclear bodies [NBs] and photobodies) as well as for binding to the promoter of and inducing the expression of the flowering inducer *FLOWERING LOCUS T* (*FT*) (Kaiserli et al., 2015). With respect to photomorphogenesis, TZP plays wavelength-specific roles: it acts as a positive regulator of hypocotyl growth in response to low blue light and a negative regulator under far-red light (Loudet et al., 2008; Perrella et al., 2018; Zhang et al., 2018). Low blue light leads to the recruitment of TZP and its interacting partner, the TF ZINC-FINGER HOMEDOMAIN10 (ZFHD10), to common promoter elements of growth-promoting and auxin-related genes (Perrella et al., 2018). By contrast, in response to far-red light, TZP acts as a positive regulator in phyA signaling by directly associating with and controlling phyA abundance and phosphorylation (Zhang et al., 2018). In addition, a recent study revealed a mutual upregulation of TZP and ELONGATED HYPOCOTYL5 (HY5), a key positive regulator of photomorphogenesis, in transducing far-red light signaling (Li et al., 2022). However, it is still unclear how TZP is involved in regulating red light signaling and whether it affects the function, stability, or nuclear localization of phyB and other red light signaling components.

PCH1 was initially discovered as an EC-associated protein operating alongside the three key EC components, ELF3, ELF4, and LUX ARRHYTHMO (LUX) to regulate circadian rhythms and hypocotyl elongation at night (Nusinow et al., 2011; Huang et al., 2016a). In the absence of any of the three key EC proteins, plants exhibit elongated hypocotyl phenotypes and insensitivity to photoperiodic regulation (Nusinow et al., 2011). *pch1* mutants exhibit daylength-specific phenotypes, with longer hypocotyls in short-day photoperiods, but not in long-day conditions (Huang et al., 2016b). Subsequent experiments revealed an interaction between PCH1 and phyB (Huang et al., 2016b), uncovering its role in red light signaling by modulating phyB photo-reversibility (Enderle et al., 2017) and NB formation (Huang et al., 2019). More specifically, PCH1 was shown to control photoperiodic hypocotyl growth by promoting phyB-dependent destabilization of PIF4. Recent studies have revealed that PCH1 inhibits the thermal reversion of phyB and acts as a structural component for phyB NB formation (Enderle et al., 2017; Huang et al., 2019).

Since affinity purification coupled with mass spectrometry (AP-MS) analysis showed that PCH1, phyB, and TZP co-precipitate (Huang et al., 2016b), these results prompted us to explore the genetic relationship between TZP and PCH1 and the role of TZP in red light signaling and photoperiodic hypocotyl elongation. This study unveils a phenotypic and molecular role for TZP in hypocotyl growth in response to red light and long days (LDs) through genetic studies using single, double, and triple mutants lacking TZP, PCH1, and/or phyB function. Our data show that PCH1 affects TZP function and localization and uncover how TZP acts as a positive regulator of phyB signaling.

## Results

### TZP and PCH1 act synergistically to regulate hypocotyl growth

**Figure 1:**
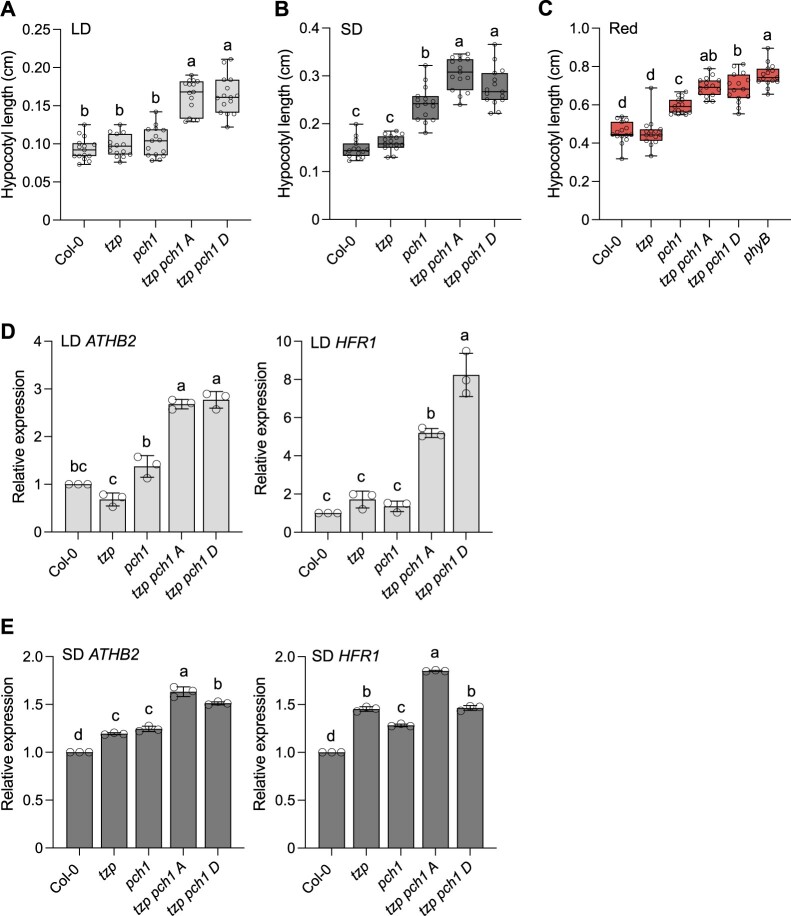
The loss of TZP function enhances the elongated hypocotyl phenotype of *pch1* in LDs, SDs, and red light. A–C, Hypocotyl measurements of mutant combinations of *tzp* with *pch1* and control lines. Seedlings were grown for 5 days in white light (60 µmol m^−2^ s^−1^) under a LD (16-h light/8-h dark) (A) or SD (8-h light/16-h dark) (B) photoperiod. C, Seedlings were grown for 5 days in red light (1 µmol m^−2^ s^−1^). Hypocotyl length was measured from digital images using ImageJ. In whisker plots, boxes show median, IQR, and maximum–minimum interval of each data set (*n* = 15 seedlings). The IQR was calculated based on the formula: quartile_3_ (*Q*_3_) – quartile_1_ (*Q*_I_). Whiskers represent *Q*_I_ – 1.5 × IQR and Q3 + 1.5 × IQR. Different lowercase letters represent significant differences by one-way ANOVA with Tukey’s post hoc test between assessed samples (*P* < 0.05). Data shown are representative of three biological replicates with independent populations of plants. D and E, RT-qPCR analysis of *ATHB2* and *HFRI* mRNA levels normalized to the housekeeping gene *ISU1* in the indicated genotypes. Tissue was harvested on the fourth day (ZT = 8 for LD, ZT = 0 for SD), *tzp pch1* A, D, indicate two independent double mutant lines. Data are means ± se of three biological replicates with independent pools of tissue. Different lowercase letters represent significant differences by one-way ANOVA with Tukey’s post hoc test (*P* < 0.05).

Quantitative MS studies showed that TZP and PCH1 are members of a multi-protein complex, with phyB acting as the central recruiting component (Huang et al., 2016a, 2016b). Although no direct interaction was reported between TZP and PCH1 (Huang et al., 2016b), we investigated the genetic relationship between these two components. For this purpose, we crossed the *tzp* and *pch1* single mutants to generate the *tzp pch1* double mutant, for which we selected two independent homozygous lines (*tzp pch1* A and *tzp pch1* D) for phenotypic analysis. We also overexpressed *TZP* in the *pch1* mutant background by both Agrobacterium (*Agrobacterium tumefaciens*)-mediated transformation and via genetic crossing to test whether *TZP* overexpression can compensate for the loss of PCH1. To assess the genetic interaction between *TZP* and *PCH1*, we performed hypocotyl elongation assays in response to short day (SD, 8-h light/16-h dark) and LD (16-h light/8-h dark) photoperiodic conditions, since PCH1 was reported to play a role in photoperiod-mediated regulation of hypocotyl growth (Huang et al., 2016b). We observed that *pch1* exhibits day length-specific defects in hypocotyl elongation (Figure 1). Under a SD photoperiod, *pch1* showed an elongated hypocotyl phenotype compared with the wild type (Col-0, WT) (Figure 1B), while *pch1* hypocotyl elongation resembled the phenotype observed in WT under LDs (Figure 1A). In response to SD, the elongated hypocotyl phenotype seen in *tzp pch1* was more pronounced compared with the *pch1* single mutant (Figure 1B). However, under two different intensities of white light in LD, the simultaneous absence of both PCH1 and TZP function led to an elongated hypocotyl phenotype not observed in the *tzp* or *pch1* single mutants (Figure 1A and Supplemental Figure S1E). Taken together, our data show that the *tzp pch1* double mutant exhibits impaired hypocotyl regulation in SD; we also uncovered a new role for TZP and PCH1 in LD-mediated hypocotyl elongation (Figure 1, A and B and Supplemental Figure S1E).

We also conducted hypocotyl elongation assays under monochromatic conditions to determine the wavelength specificity of the afore-mentioned photoperiod-mediated hypocotyl growth responses. In line with previous reports, *pch1* seedlings showed an elongated hypocotyl compared with WT in response to three different intensities of red light (Figure 1C and Supplemental Figure S1, C, D, G, and H), while blue, far-red light, and darkness had no effect on the hypocotyl length of *pch1* (Supplemental Figure S1, A, B, and F), indicating that PCH1 is a positive regulator of red light-mediated photomorphogenesis (Huang et al., 2016b). Introducing the *tzp* mutation into the *pch1* mutant background led to a more pronounced hypocotyl elongation in response to red light, as the two *tzp pch1* double mutant lines had an elongated hypocotyl comparable to that displayed in the null *phyB-9* mutant (Figure 1C). Both *tzp pch1* double mutant lines showed statistically significant elongated hypocotyls compared with WT and *pch1* under far-red light (Supplemental Figure S1B), which also suggested that PCH1 may play a role in the far-red light signaling pathway along with TZP. These findings indicate that red light-controlled photomorphogenesis is disrupted in the absence of both TZP and PCH1 function and that TZP and PCH1 together play indispensable roles in the red-light signaling pathway.

To understand the molecular mechanism underlying the new and additive phenotype *of tzp pch1* under LD and SD conditions, respectively, we performed reverse transcription quantitative PCR (RT-qPCR) to monitor the expression of hypocotyl elongation marker genes operating downstream of phyB such as *ARABIDOPSIS THALIANA HOMEOBOX PROTEIN 2* (*ATHB2*) and *LONG HYPOCOTYL IN FAR-RED* (*HFR1*) (Huang et al., 2016b). The induction of *ATHB2* and *HFR1* expression is positively correlated with hypocotyl elongation in low light conditions (Kunihiro et al., 2011; Pedmale et al., 2016; Perrella et al., 2018). The expression levels of both *ATHB2* and *HFR1* increased in *tzp pch1* compared with WT, *tzp*, and *pch1* (Figure 1, D and E). We concluded that the elongated hypocotyl phenotype observed in *tzp pch1* can be partially attributed to the upregulated expression of *ATHB2* and *HFR1* in SD and LD. *ATHB2* is known to be a target locus for multiple light (shade, blue, red light), phytohormone (auxin), and clock pathways regulating hypocotyl growth; therefore, the *ATHB2* expression pattern observed in the *tzp* and *pch1* single mutants may be masked or compensated by other factors.

**Figure 2:**
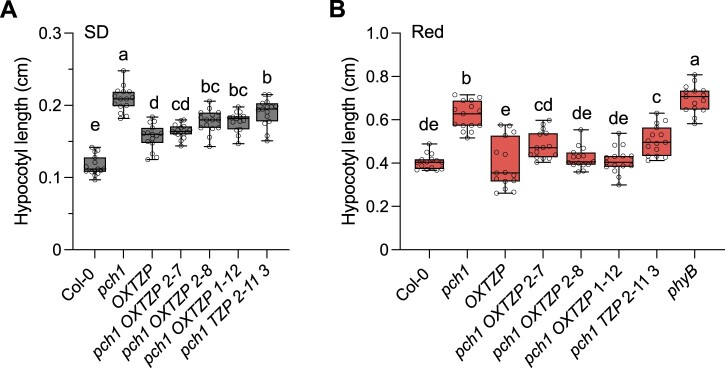
Overexpression of *TZP* counteracts the *pch1* phenotype in SDs and red light. A and B, Hypocotyl measurements of Col-0, *OXTZP*, *pch1*, *pch1 OXTZP* lines (2–7, 2–8, 1–12), the *pch1 TZP* (2–11 3) transgenic line, and *phyB-9*. Seedlings were grown for 5 days in white light (60 µmol m^−2^ s^−1^) SD conditions (8-h light/16-h dark) (A) or constant red light (1 µmol m^−2^ s^−1^) (B). Data shown are representative of three biological replicates with independent populations of seedlings. Hypocotyl length was measured from digital images using ImageJ. In whisker plots, boxes show median, IQR, and maximum–minimum interval of each data set (*n* = 15 seedlings). Different lowercase letters represent significant differences by one-way ANOVA with Tukey’s post hoc test among assessed samples (*P* < 0.05).

To further elucidate the relationship between TZP and PCH1, we generated transgenic lines overexpressing *TZP-GFP* (referred to as *OXTZP*) in *pch1* and performed genetic crosses between *OXTZP* (Kaiserli et al., 2015) and *pch1*. We examined TZP abundance in the afore-mentioned transgenic lines and genetic crosses by immunoblotting; we selected independent lines showing TZP-GFP protein levels comparable to the previously characterized *OXTZP* line (Loudet et al., 2008; Kaiserli et al., 2015; Supplemental Figure S2, F and H) for further analysis (hypocotyl elongation assays, sub-cellular localization). We established that the elongated hypocotyl phenotype of *pch1* is suppressed by the overexpression of *TZP* under SD (Figure 2A). *pch1 OXTZP* lines showed a partial reversal of the *pch1* phenotype, indicating that *TZP* overexpression can rescue the elongated hypocotyl phenotype of *pch1* in constant red light, or that OXTZP potentially acts epistatically to PCH1 (Figure 2B). On the contrary, *pch1 OXTZP* showed a hypocotyl elongation response similar to that of Col-0 and *pch1* in blue light (Supplemental Figure S2B), suggesting that PCH1 may be required for TZP-mediated hypocotyl growth in blue light (Loudet et al., 2008; Perrella et al., 2018). These results further support our findings that the LD-specific phenotype observed in *tzp pch1* is due to the combined action of mutations in *TZP* and *PCH1*.

### TZP affects phyB and PIF4 abundance in LDs and red light

**Figure 3:**
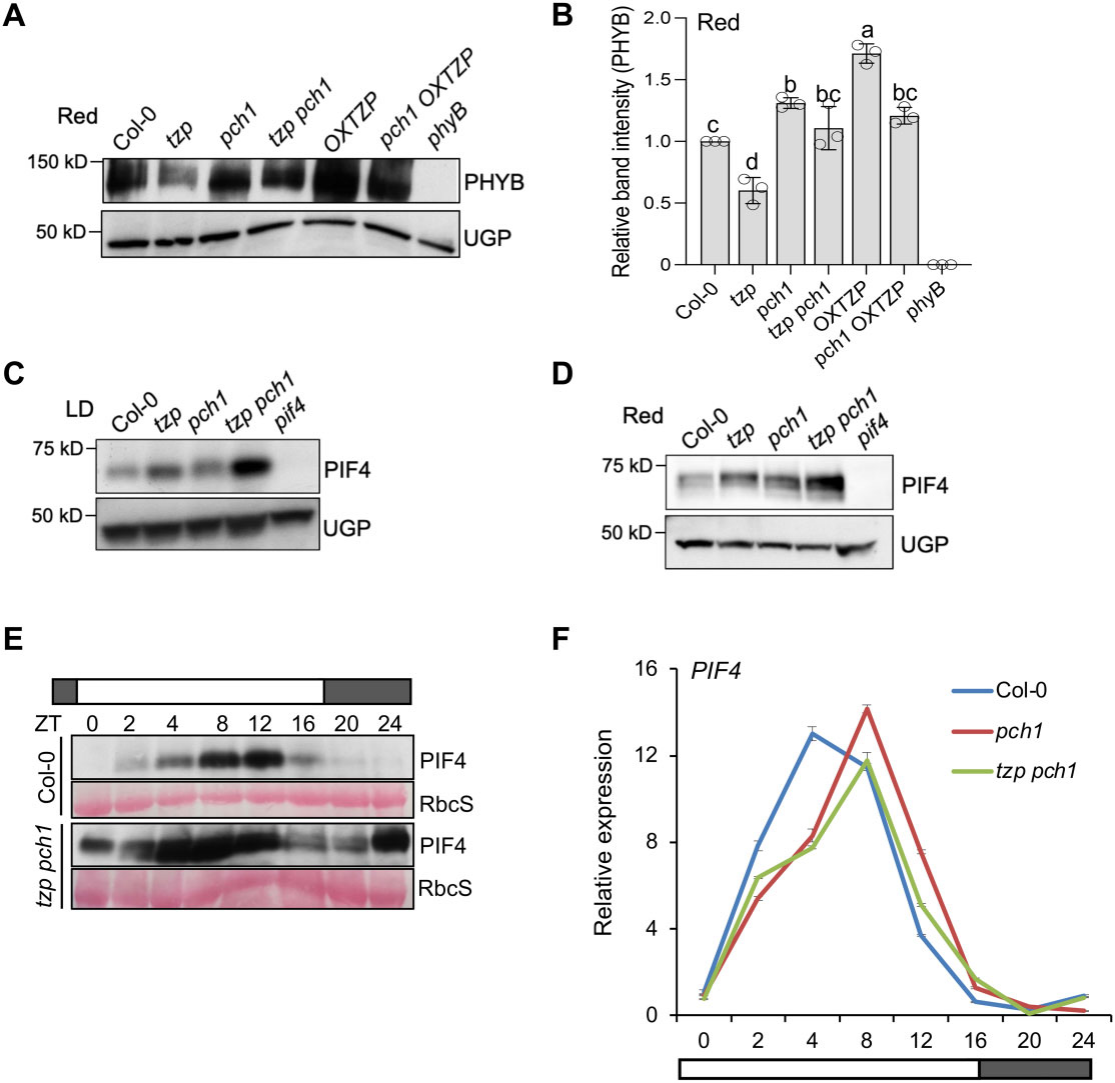
PIF4 protein levels increase in *tzp pch1*. A, Immunoblot analysis of phyB protein levels from seedlings grown in continuous red light (1 µmol m^−2^ s^−1^) for 4 days. An anti-phyB antibody was used to detect phyB protein and anti-UGP to monitor UGP as loading control. The *phyB-9* mutant was used as a negative control. B, Quantification of the relative band intensities (phyB/UGP) of the immunoblot shown in (A), as performed in ImageJ. Data are means ± sd from three biological replicates with independent pools of tissue. C and D, Immunoblot analysis of PIF4 protein levels from seedlings grown in LD conditions (white light 60 µmol m^−2^ s^−1^) (C), or continuous red light (1 µmol m^−2^ s^−1^) (D) for 4 days. Protein was isolated at ZT8 (8 h after lights on) in LD. PIF4 protein levels were detected by an anti-PlF4-specific antibody. UGP was used as loading control. A *pif4* mutant was used as a negative control. Data shown are representative of three biological replicates with independent pools of tissue. Quantification of relative band intensities of (C and D) can be found in Supplemental Figure S3, B and C. E, Time course analysis of PIF4 protein levels from seedlings grown in a LD photoperiod (white light 60 µmol m^−2^ s^−1^) for 10 days. Tissue was harvested every 4 h from ZT0, with an extra time point at ZT2. Tissue collection at ZT0 and ZT24 was conducted in darkness. PIF4 protein levels were determined with an anti-PlF4-specific antibody. Ponceau S staining of the housekeeping protein RbcS (small subunit of rubisco) was used as loading control. F, Time course analysis of *PIF4* transcript levels from seedlings grown in identical conditions as in (E). *PIF4* transcript levels were normalized to the housekeeping gene *ISU1*. Data shown are representative of three biological replicates with independent pools of tissue.

Since the *tzp pch1* double mutant suppressed red light signaling with regards to controlling hypocotyl elongation (Figure 1C), we tested whether altered phyB protein abundance is accountable for the impaired red-light response in *tzp pch1*. Immunoblot analysis using an anti-phyB antibody showed that the red-light-specific hypocotyl elongation phenotype observed in *tzp pch1* is not due to a decrease in total phyB protein levels, as *tzp pch1* showed phyB protein abundance comparable to WT (Figure 3, A and B). However, *OXTZP* showed an increase in phyB protein levels, while *tzp* exhibited a decrease in phyB abundance compared with WT (Figure 3, A and B), suggesting that TZP may stabilize phyB protein during prolonged red-light exposure. Nevertheless, this TZP-dependent phyB accumulation was impaired in *pch1 OXTZP* (Figure 3, A and B), indicating that PCH1 may regulate the influence of TZP on phyB protein.

Since a reduction in phyB protein levels did not account for the reduced red-light response observed in *tzp pch1*, we investigated alternative mechanisms by focusing on signaling components acting downstream of phyB. PIF4 abundance (Huq and Quail, 2002) directly correlates with phyB-mediated hypocotyl elongation phenotypes (Huang et al., 2016b; Park et al., 2018). We therefore asked whether altered PIF4 levels are the underlying cause for the difference in hypocotyl length between *pch1* and *tzp pch1*. Accordingly, we performed RT-qPCR and immunoblot analysis to monitor *PIF4* mRNA and PIF4 protein abundance in *tzp*, *pch1*, and *tzp pch1* in LD conditions and in constant red light. We collected tissue at Zeitgeber 8 (ZT8, 8 h after lights on) in LD, when *PIF4* shows a peak in transcript levels that matches the peak in hypocotyl growth (Nozue et al., 2007). Relative *PIF4* transcript levels were not significantly altered in *tzp pch1* compared with *pch1* in LD or red light (Supplemental Figure S3, D and E). By contrast, under LD photoperiodic conditions, *tzp pch1* showed increased PIF4 protein levels compared with *pch1* and WT, while *pch1* had PIF4 levels similar to WT (Figure 3C and Supplemental Figure S3B). These data were in line with the hypocotyl phenotype of *tzp pch1* in LD (Figure 1A) and further supported our conclusion that an increase in PIF4 protein abundance may be the underlying reason for the elongated hypocotyl phenotype observed in *tzp pch1* in LDs. We also observed an increase in PIF4 accumulation in *tzp pch1* compared with *pch1* in red light (Figure 3D and Supplemental Figure 3C), which correlates with the hypocotyl phenotype seen under this condition (Figure 1C).

To further explore the molecular mechanism underlying the LD-specific hypocotyl phenotype exhibited by *tzp pch1*, we conducted a more detailed analysis of *PIF4* transcript and PIF4 protein levels using time course experiments. To this end, we grew WT and *tzp pch1* seedlings for 10 days in LD and collected tissue every 4 h for 24 h, starting at ZT0 on day 11 after germination with an extra time point at ZT2. PIF4 accumulation followed similar diurnal rhythms in WT and *tzp pch1*, exhibiting a peak during daytime in LD (Nomoto et al., 2013), although PIF4 was much more abundant in *tzp pch1* compared with WT during the entire time course (Figure 3E). By contrast, relative *PIF4* transcript levels followed a similar pattern in all three genotypes (Col-0, *pch1*, and *tzp pch1*), except for an increase in *PIF4* mRNA at ZT4 in WT (Figure 3F). These results were consistent with the data collected from single time point experiments (Figure 3C and Supplemental Figure S3D), suggesting that an increase in PIF4 protein is predominantly accountable for the elongated hypocotyl phenotype exhibited by *tzp pch1* in LD and red light (Figure 1, A and C). However, we cannot exclude the possibility that TZP also regulates PIF4 at the transcriptional level but to a lesser extent.

### PCH1 plays a role in TZP NB formation

**Figure 4:**
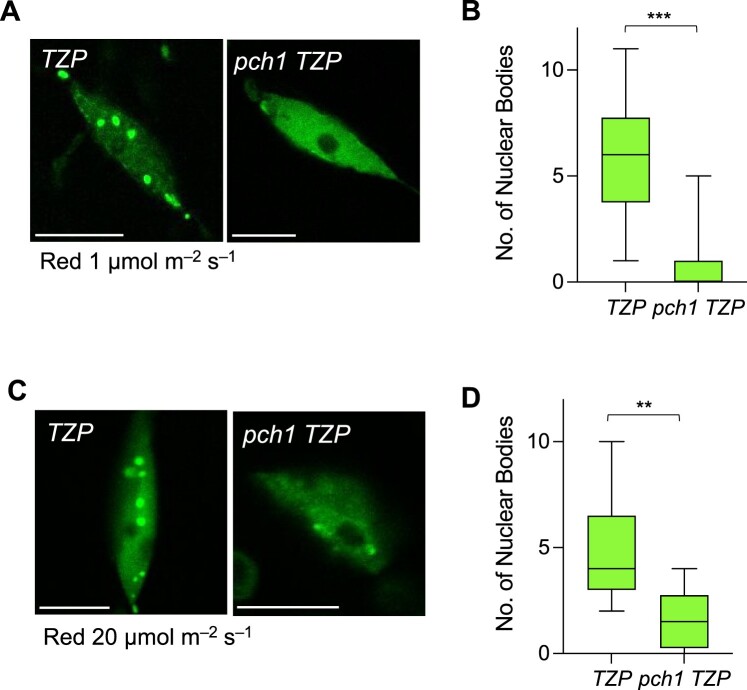
TZP nuclear body formation is impaired in *pch1*. A and C, Confocal image analysis of nuclear body (NB) abundance of GFP-tagged TZP (*OXTZP*) and a *pch1 TZP* (lines 1–12) crossing line. Representative images of the outermost epidermal cells of the upper third hypocotyl part of 4-day-old etiolated seedlings exposed to red light (A) 1 µmol m^−2^ s^−1^ or (C) 20 µmol m^−2^ s^−1^ for 24 h are shown. Scale bars, 10 µm. B and D, Quantification analysis of the number of NBs per nucleus from confocal images of *TZP* and *pch1 TZP* in response to the conditions described in (A) and (C), respectively. Confocal images were analyzed in ImageJ and graphs were plotted in GraphPad Prism. In whisker plots, boxes show median, IQR, and maximum–minimum interval of each data set. A minimum of 12 nuclei per condition and per biological replicate were examined. Asterisks (**) represent *P* < 0.01 by one-way ANOVA and indicate a significant difference compared with *TZP*. Data shown are representative of three biological replicates with independent populations of seedlings.

In addition to co-associating and regulating light and photoperiodic hypocotyl growth, TZP, PCH1, and phyB have been reported to localize to NBs, also known as photobodies, in response to red and white light (Loudet et al., 2008; Kaiserli et al., 2015; Huang et al., 2016b; Enderle et al., 2017). TZP is recruited to NBs through a direct interaction with phyB (Kaiserli et al., 2015), whereas PCH1 has been shown to act as a structural component required for phyB NB formation and stability (Enderle et al., 2017; Huang et al., 2019). To assess if PCH1 regulates TZP localization, we examined the formation of TZP NBs in WT or the *pch1* mutant overexpressing a construct encoding a fusion between TZP and the green fluorescent protein (*TZP-GFP*, *OXTZP* thereafter) (Figure 4). To eliminate the possibility of impaired TZP NB formation due to a difference in total TZP protein levels, we monitored TZP–GFP protein levels in WT and *pch1* by immunoblot analysis (Supplemental Figure S2, F–I). We then selected independent lines showing comparable levels of TZP–GFP in WT and *pch1* for quantitative analysis of TZP NB formation (Supplemental Figure S2, F–I). Accordingly, we employed confocal microscopy to monitor the abundance and morphology of TZP–GFP NBs in *OXTZP* and *pch1 OXTZP* hypocotyl cells in response to two different fluence rates of red light (1 and 20 μmol m^−2^ s^−1^) for 24 h, as shown in Figure 4. NB formation is closely correlated to the wavelength, intensity, and duration of light (Van Buskirk et al., 2014). The formation of TZP NBs occurs rapidly in response to red light (Kaiserli et al., 2015). We hypothesized that the lower number of TZP NBs seen in *pch1 OXTZP* (Figure 4 and Supplemental Figure S4B) is due to either: (1) an impairment of TZP NB formation or (2) a faster dissociation of TZP NBs in the absence of PCH1. To distinguish between these two possibilities, we performed a quantitative analysis of the total number of NBs (Figure 4, B and D) or nuclei harboring large NBs (>0.6 µm^2^) (Supplemental Figure S4, A and B). This method was adapted from a previous study on phyB NB formation (Huang et al., 2019). Our data revealed that the loss of functional PCH1 severely impairs the formation of TZP NBs after a 24-h low red-light treatment (1 μmol m^−2^ s^−1^) (Figure 4B) and after a 24-h red light exposure at a higher fluence rate (20 μmol m^−2^ s^−1^), with over 60% of the nuclei showing five or more NBs in *OXTZP*, and around 13% nuclei harboring over five NBs in *pch1 OXTZP* (Figure 4C and Supplemental Figure S4A). These results confirmed that TZP NB formation is impaired in the *pch1* background. A transition from red light to darkness revealed a decrease in NB number in *pch1 OXTZP* compared with *OXTZP* after a 1-h dark incubation (Supplemental Figure S4B), which could be attributed to the decrease in the total number of NBs of red light-exposed *pch1 OXTZP* seedlings prior to the dark treatment.

Overall, Figure 4 showed that the loss of PCH1 impairs TZP NB formation. These results also support the conclusion that NBs have functional significance (Van Buskirk et al., 2014), as the fewer NBs formed in *pch1 OXTZP* correlated with shorter hypocotyl phenotypes in this line compared with *OXTZP* in LDs (Supplemental Figure S2A) and blue light (Supplemental Figure S2B). The finding that PCH1 is required for proper TZP NB formation is consistent with the role of PCH1 as a structural component of phyB NBs and that TZP is recruited to NBs by phyB (Kaiserli et al., 2015; Huang et al., 2019).

### TZP modulates phyB protein abundance and photobody morphology

**Figure 5:**
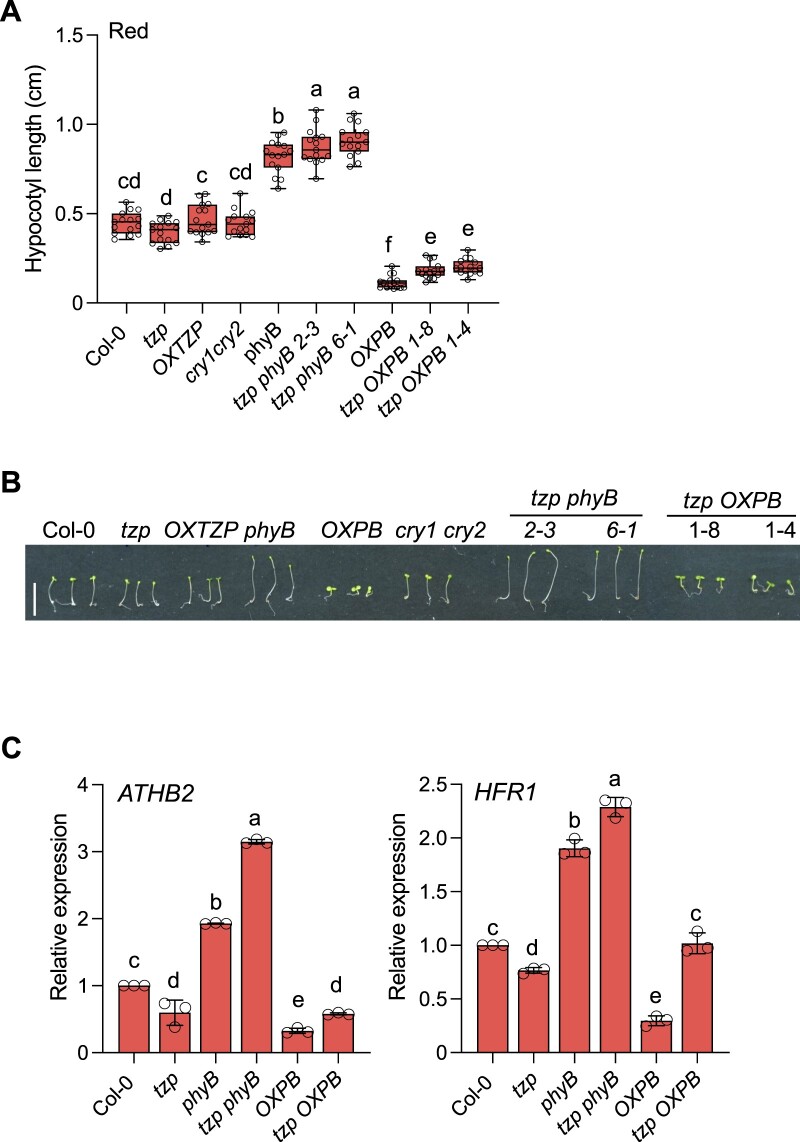
TZP regulates phyB-mediated hypocotyl elongation and gene expression in red light. A and B, Hypocotyl measurements and phenotypes of mutant combinations between *tzp* and *phyB-9* or *phyBOX* (*OXPB*) and the corresponding parental lines. Surface-sterilized and stratified seeds were grown for 5 days in red light (1 µmol m^−2^ s^−1^); seedlings were scanned at the end of the fifth day. Hypocotyl length was measured from digital images using ImageJ. Graph of hypocotyl measurements (A) and representative image (B) of Col-0, *tzp*, *OXTZP*, *phyB-9*, *OXPB*, *tzp phyB* 2-3, *tzp phyB* 6-1, *tzp OXPB* 1-8, and *tzp OXPB* 1-4 seedlings grown in the indicated conditions (scale bar, 5 mm) are shown. In whisker plots, boxes show median, IQR, and maximum–minimum interval of each data set (*n* = 15 seedlings). Data shown are representative of three biological replicates with independent populations of seedlings. Different lowercase letters represent significant differences by one-way ANOVA with Duncan’s post hoc test between assessed samples (*P* < 0.05). C, RT-qPCR analysis of *ATHB2*, *HFR1* transcript levels, normalized to the housekeeping gene *ISU1* in the indicated genotypes. Surface-sterilized and stratified seeds were first exposed to white light (60 µmol m^−2^ s^−1^) for 5 h before being exposed to 5 µmol m^−2^ s^−1^ red light; tissue was collected on the fifth day after red light exposure. Data are means ± se of three biological replicates with independent pools of tissue. Different lowercase letters represent significant differences by one-way ANOVA with Tukey’s post hoc test (*P* < 0.05).

As our data indicate that TZP plays a role in PCH1-mediated signaling (Figure 1C), we decided to reexamine a potential function for TZP in red light-mediated hypocotyl elongation, even though no obvious phenotype had been reported for *OXTZP* or *tzp* in red light (Kaiserli et al., 2015; Perrella et al., 2018; Zhang et al., 2018). More specifically, we investigated the genetic interaction between TZP and phyB by examining the hypocotyl phenotypes of the following lines generated by genetic crossing between *tzp* and *phyB-9* (*tzp phyB*) or *tzp* and *35Spro*:*PHYB-CFP* (*tzp OXPB*, encoding a fusion between phyB and the cyan fluorescent protein [CFP]). We observed that the *tzp phyB* double mutant lines exhibit red light-mediated hypocotyl elongation responses similar to those of *phyB-9* (Figure 5A). These findings indicated that *phyB-9* is epistatic to *tzp* in response to red light, which is in accordance with previously published data (Zhang et al., 2018). The overexpression of *PHYB* (*OXPB*) results in extremely short hypocotyls caused by enhanced sensitivity to red light and increased inhibition of hypocotyl elongation (Figure 5A; Wagner et al., 1991; Nito et al., 2013). Overexpression of *PHYB* in *tzp* (*tzp OXPB1* #8 and *tzp OXPB1* #4) led to slightly longer hypocotyls than those of the parental *OXPB* line (*P *<* *0.001), indicating reduced sensitivity to red light (Figure 5A). These results suggested that although native TZP shows no significant regulation of hypocotyl growth under red light, the loss of TZP may hinder phyB function in regulating hypocotyl growth inhibition under conditions of high phyB levels. More specifically, the role of *OXPB* in inhibiting red light-mediated hypocotyl growth is impaired in the absence of TZP function.

In response to blue light, lines overexpressing *TZP* (*OXTZP*) led to an elongated hypocotyl phenotype, while *tzp* showed a shorter hypocotyl, as previously reported (Kaiserli et al., 2015; Perrella et al., 2018). Although *phyB-9* mutant seedlings resembled Col-0 in blue light, *tzp phyB* double mutant seedlings showed an unanticipated elongated hypocotyl phenotype, which may indicate a role for TZP in bridging red light and blue light signaling by associating with phytochromes and cryptochromes separately and mediating signal integration (Supplemental Figure S5A). We tested hypocotyl growth under darkness to minimize the effect of nonspecific growth defects or germination inconsistencies; all genotypes had comparable etiolated hypocotyl lengths (Supplemental Figure S5B).

To better understand the molecular mechanism underlying the physiological response of *tzp phyB and tzp OXPB* to the light conditions examined, we monitored the relative transcript levels of the TZP and phyB-regulated TF genes *ATHB2* and *HFR1* by RT-qPCR. *ATHB2* and *HFR1* expression was lower in *OXPB* than in WT, in agreement with the hypersensitive response to red light displayed by *OXPB*. Nevertheless, the expression of these genes was significantly upregulated in *tzp OXPB* compared with the parental *OXPB* line (Figure 5C) in response to red light. These data indicated that *OXPB* is less functional in suppressing the expression of these growth-promoting genes in the absence of TZP function, resulting in an elongated hypocotyl phenotype in *tzp OXPB* relative to *OXPB* (Figure 5A). By contrast, low blue light illumination resulted in higher expression levels for *ATHB2* and *HFR1* in *tzp phyB* compared with *phyB-9* or Col-0 seedlings (Supplemental Figure S5, C and D), consistent with the hypocotyl phenotypes observed in these lines in response to blue light (Supplemental Figure S5A).

### TZP stabilizes phyB protein

**Figure 6:**
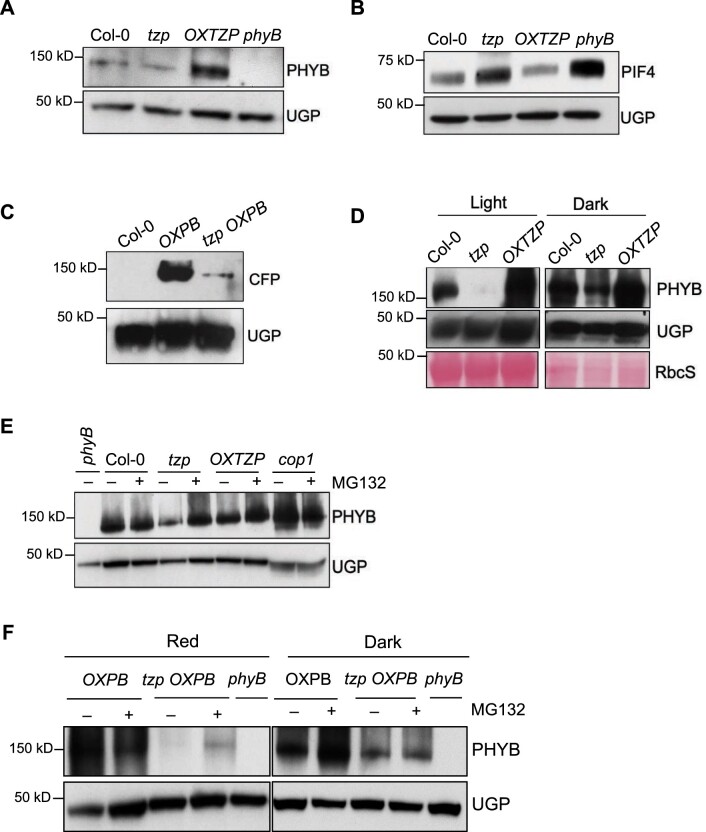
TZP stabilizes phyB protein. A, Immunoblot analysis of phyB protein levels from seedlings grown in constant red light (1 µmol m^−2^ s^−1^) for 4 days by using an anti-phyB-specific antibody. UGP was used as a loading control. B, Immunoblot analysis of PIF4 protein levels of seedlings grown under the same conditions as in (A). PIF4 protein levels were determined with an anti-PlF4-specific antibody; UGP was used as loading control. Quantification of relative band intensities (A–B) can be found in Supplemental Figure S6, A and B. C, Immunoblot analysis of CFP-tagged phyB protein in *OXPB* and *tzp OXPB* grown under the same conditions as in (A). An anti-GFP antibody was used to detect CFP-tagged phyB in *OXPB* and *tzp OXPB*. UGP was used as loading control and Col-0 was used as a negative control. D, Immunoblot analysis of phyB protein levels in 4-day-old seedlings grown in constant white light (60 µmol m^−2^ s^−1^) or kept in darkness. An anti-phyB-specific antibody was used to detect phyB protein. UGP and a Ponceau S stain of RbcS were used as loading controls. Quantification of the relative band intensities can be found in Supplemental Figure S6E. E, Immunoblot analysis of phyB protein levels in 4-day-old etiolated seedlings infiltrated with 50 µM of the proteasomal inhibitor MG132 or an equal volume of DMSO in liquid half-strength MS medium for 2 h in the dark prior to a 2-h red light (25 µmol m^−2^ s^−1^) exposure. Quantification of the relative band intensities can be found in Supplemental Figure S6F. F, Four-day-old etiolated seedlings were infiltrated with 50 µM of the proteasomal inhibitor MG132 or DMSO in liquid half-strength MS medium for 2 h in the dark prior to a 2-h red light (25 µmol m^−2^ s^−1^) exposure or kept in darkness. Quantification of the relative band intensities (F) can be found in Supplemental Figure S6G. PhyB protein levels were determined with an anti-phyB-specific antibody. UGP was used as loading control. The *phyB-9* mutant was used as negative control. Data shown are representative of three biological replicates with independent pools of tissue.

To further understand how TZP modulates phyB activity, we tested a series of hypotheses. Our first hypothesis posits that TZP may regulate phyB transcriptional or post-transcriptional abundance. Inspired by the work showing an increase in phyA protein accumulation in *tzp* seedlings in response to far-red light (Zhang et al., 2018) and the results demonstrated in Figure 3A, we monitored native phyB protein abundance in WT, *tzp*, and *OXTZP* seedlings in response to red light by immunoblot analysis using an anti-phyB antibody (Figure 6A). We observed that phyB protein levels are higher in *OXTZP* and lower in *tzp* compared with WT in 4-day-old red light-grown seedlings (Figure 6A). These data indicated that phyB accumulation correlates with the presence or absence of TZP. More specifically, TZP promoted phyB protein accumulation. We also verified our observation that *OXTZP* leads to a decrease in PIF4 protein abundance, and *tzp* to an increase in PIF4 protein levels compared with WT in seedlings exposed to red light (Figure 6B). This discovery directly correlated with the effect of TZP on phyB protein abundance, which was in accordance with previous studies showing that phyB negatively regulates PIF4 protein stability (Leivar et al., 2008). Although we observed significant differences in endogenous phyB protein levels between *OXTZP* and *tzp* (Supplemental Figure S6), the effect on hypocotyl elongation was negligible in red light (Figure 5A). We therefore performed an immunoblot analysis on *OXPB* and *tzp OXPB* for phyB levels, since these lines displayed statistically significant phenotypic changes in response to red light (Figure 6C). Indeed, phyB protein levels were much lower in *tzp OXPB* compared with *OXPB* (Figure 6C). Therefore, it is highly likely that the phenotypic difference with respect to hypocotyl elongation observed in *OXPB* and *tzp* OXPB (Figure 5A) could be due to the strong reduction in phyB protein abundance seen in *tzp OXPB* compared with *OXPB* (Figure 6C).

We also determined relative *PHYB* transcript levels in *tzp*, *OXTZP*, *OXPB*, *tzp OXPB*, and WT seedlings exposed to identical light treatments to the ones described in the immunoblot experiments above (Supplemental Figure S6, C and D); we detected no significant difference in *PHYB* mRNA levels between WT and *tzp*, while *OXTZP* showed slightly lower *PHYB* expression than WT (Supplemental Figure 6C). Likewise, we observed no significant difference in *PHYB* mRNA in *tzp OXPB* compared with *OXPB* (Supplemental Figure S6D). Thus, the possibility that a transcriptional mechanism may underlie the observed change in phyB protein levels among genotypes is negligible. We conclude that TZP predominantly regulates phyB action at the protein level.

Our next hypothesis explored whether TZP interferes with phyB degradation. Photoactivated phyB translocates to the nucleus and undergoes proteasomal degradation (Clough and Vierstra, 1997; Jang et al., 2010). To test whether TZP contributes to phyB degradation, we performed an immunoblot analysis on seedlings grown in the dark or in constant light. We determined that *tzp* is characterized by a decrease in phyB protein levels compared with WT in darkness (Figure 6D). We observed a more dramatic reduction in phyB abundance in *tzp* growing in constant light, with phyB signal being almost undetectable at the indicated exposure time. These results validated the hypothesis that phyB undergoes a more severe degradation in the absence of TZP than in WT primarily in response to light, whereas degradation was reduced in *OXTZP* (Figure 6D). To further investigate whether TZP leads to an increase in phyB protein abundance by interfering with its proteasomal degradation, we employed the proteasomal inhibitor MG132 (Figure 6, E and F). As phyB degradation is primarily mediated by CONSTITUTIVELY PHOTOMORPHOGENIC1 (COP1), an E3 ubiquitin ligase (Jang et al., 2010; Ni et al., 2014), we used the *cop1-4* mutant as a negative control for this experiment. We thus applied MG132 to WT, *tzp*, and *OXTZP* seedlings to monitor how endogenous phyB levels change. We also treated *OXPB* and *tzp OXPB* seedlings with MG132 to test whether phyB-CFP degradation is altered in the presence or absence of TZP. We established that phyB degradation is more prominent in *tzp* than in WT, as we observed a significant accumulation of phyB protein after the MG132 treatment, whereas the application of MG132 led to a more moderate effect on the accumulation of phyB protein in WT and *OXTZP* (Figure 6E and Supplemental Figure S6F). We observed a similar trend in *OXPB* and *tzp OXPB*, whereby *tzp OXPB* showed an increase in phyB abundance upon MG132 treatment in red light (Figure 6F and Supplemental Figure S6G). On the contrary, etiolated *tzp OXPB* seedlings showed similar phyB protein accumulation regardless of MG132 treatment. These observations support our hypothesis that TZP may protect phyB from red light-mediated proteasomal degradation.

### TZP modulates phyB signaling

**Figure 7:**
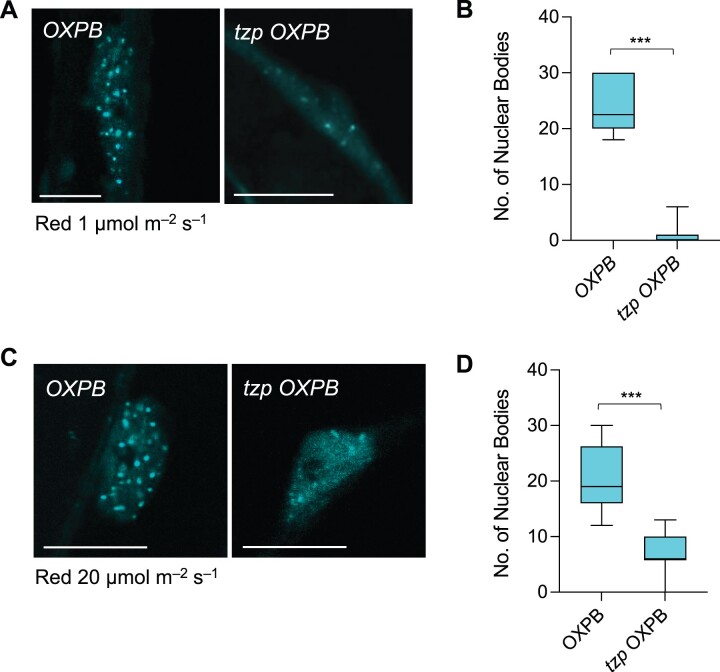
TZP regulates phyB nuclear body formation. A and C, Confocal image analysis of nuclear body (NB) abundance of CFP-tagged phyB (*OXPB*) and *tzp OXPB*. Representative images of the outermost epidermal cells of the upper third hypocotyl part of 4-day-old etiolated seedlings exposed to 1 µmol m^−2^ s^−1^ (A) or 20 µmol m^−2^ s^−1^ (C) red light for 24 h are shown. Scale bars, 10 µm. B and D, Quantification analysis of the number of NBs per nucleus from confocal images of *OXPB* and *tzp OXPB* in response to the conditions described in (A) and (C), respectively. Confocal images were analyzed in ImageJ and graphs were plotted in GraphPad Prism. In whisker plots, boxes show median, IQR, and maximum/minimum interval of each data set. A minimum of 12 nuclei per condition and per biological replicate were examined. Asterisks (***) represent *P* < 0.001 by one-way ANOVA and indicate a significant difference compared with *OXPB*. Data shown are representative of three biological replicates with independent populations of seedlings.

**Figure 8:**
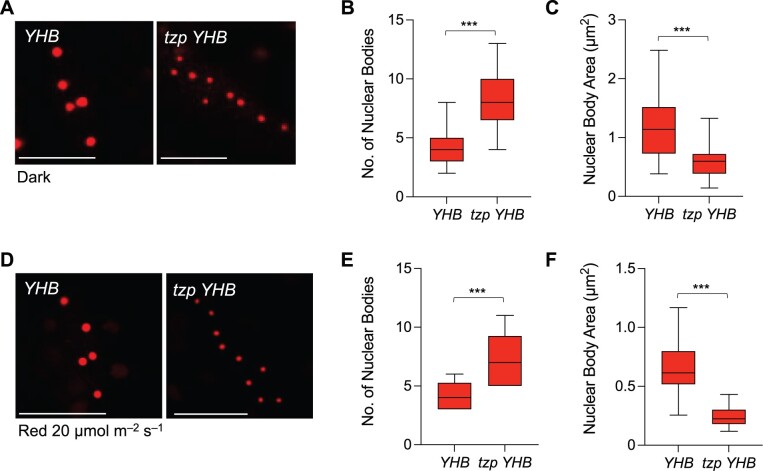
TZP regulates the nuclear body morphology and abundance of the constitutively active phyB^Y276H^. A and D, PhyB^Y276H^ (YHB) nuclear body (NB) abundance and morphology were monitored by confocal microscopy. Representative images of the outermost epidermal cells of the upper third hypocotyl part of 4-day-old seedlings grown in the dark (A) or in red light (20 µmol m^−2^ s^−1^) (D) in *YHB* and *tzp YHB* are shown. Scale bars, 10 µm. Quantification of the NB number per nucleus (B, E) and NB area (C, F) from confocal images of *YHB* and *tzp YHB* in response to the conditions described in (A) and (D), respectively. Confocal images were analyzed in ImageJ and graphs were plotted in GraphPad Prism. In whisker plots, boxes show median, IQR, and maximum–minimum interval of each data set. A minimum of 12 nuclei and per biological repeat were examined. Asterisks (***) represent *P* < 0.001 by one-way ANOVA and indicate significant difference compared with *YHB*. Data shown are representative of three biological replicates with independent populations of seedlings.

Besides the role of TZP in stabilizing phyB protein, we wished to study whether TZP affects phyB-mediated light signaling directly. phyB localizes to NBs upon red light exposure and the regulation of phyB NB kinetics reveals vital information about how red light signaling is transduced (Chen et al., 2005; Van Buskirk et al., 2014). We therefore explored the subnuclear localization pattern of phyB in *OXPB* and *tzp OXPB* by confocal microscopy. We demonstrated that phyB NB formation is impaired in *tzp OXPB* in comparison to *OXPB* (Figure 7). More specifically, *tzp OXPB* exhibited fewer NBs compared with *OXPB*. Indeed, *OXPB* formed an average of 20–25 NBs per nucleus in response to red light (1 or 20 µmol m^−2^ s^−1^) exposure for 24 h (Figure 7). By contrast, *tzp OXPB* displayed significantly fewer NBs per nucleus (one on average under low red light and up to seven at a higher red light fluence rate) compared with *OXPB*. However, we cannot rule out the possibility that this difference is due to the decrease in phyB protein levels in the absence of TZP (Figure 6C). To further examine the influence of TZP on phyB NB formation, we crossed *tzp* and a transgenic line expressing the constitutively active phyB variant *PHYB^Y276H^* (referred to as *YHB*). YHB carries a Y276H mutation in the phyB chromophore attachment domain and maintains phyB in the active Pfr form regardless of the light conditions (Su and Lagarias, 2007). PHYB^Y276H^ is also strongly fluorescent, allowing for its localization without any added fluorescent protein. The *YHB* line displayed large and stable NBs in dark-grown seedlings (Figure 8A), which was consistent with previous research (Su and Lagarias, 2007; Huang et al., 2019). Notably, we noticed an alteration in the pattern of YHB localization to NBs in *tzp YHB*. Specifically, YHB maintained its localization to NBs in the dark (Figure 8A), with NBs forming in all hypocotyl nuclei examined, most of which (>80%) contained three to five NBs per nucleus with an average area of 1.2 µm^2^ (Figure 8, B and C). In *tzp YHB*, YHB NBs still formed, but the number of NBs per nucleus increased to an average of 8 (Figure 8B). Furthermore, the average area of individual NBs in *tzp YHB* was 0.6 µm^2^ (Figure 8C), thus smaller than in *YHB*. Together, these results demonstrate that the NB formation pattern is altered in *tzp YHB*, exhibiting a higher number of smaller NBs in darkness compared with the *YHB* line (Figure 8, B and C), which was classified as the “immature” forming stage of NBs (Chen et al., 2003). We also observed a similar pattern in YHB NB imaging studies conducted on seedlings grown in red light (20 µmol m^−2^ s^−1^) (Figure 8D), where the absence of functional TZP led to an increase in the number of YHB NBs per nucleus (Figure 8E) and a decrease in their surface area compared with *YHB* (Figure 8F). To be more specific, the introduction of the *tzp* mutation partially dampened the ability of YHB to form NBs. We detected similar phyB levels in *YHB* and *tzp YHB* (line 1.7.3) (Supplemental Figure S8, A and B), strengthening the conclusion that the difference in NB formation between these two lines was not due to variation in phyB protein levels. These results indicate that TZP is involved in modulating YHB NB formation and supports the hypothesis that TZP affects phyB signaling and consequently PIF4 protein abundance. This notion would potentially provide an additional role for TZP in phyB regulation of NB formation on top of its effect on phyB protein abundance.

### TZP acts upstream of PIF4 in red light signaling

**Figure 9:**
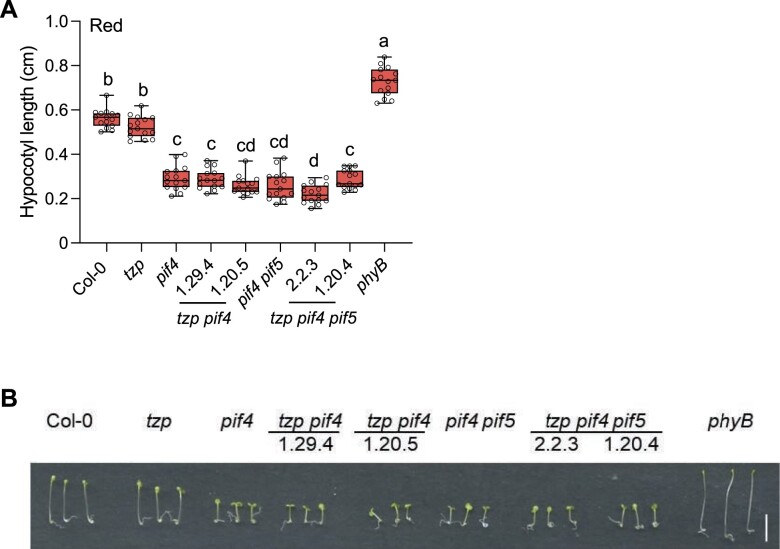
PIF4 acts downstream of TZP in red light signaling. Hypocotyl measurements of mutant combinations between *tzp* and *pif4*, *pif4 pif5* and controls. Seedlings were grown for 5 days in red light (1 µmol m^−2^ s^−1^). Hypocotyl lengths (A) and graphs of representative images (B) of Col-0, *tzp*, *pif4*, *tzp pif4* 1.29.4, *tzp pif4* 1.20.5, *pif4 pif5*, *tzp pif4 pif5* 2.2.3, *tzp pif4 pif5* 1.20.4, *phyB* seedlings grown in the indicated conditions. *tzp pif4* 1.29.4, *tzp pif4* 1.20.5 are two independent *tzp pif4* double mutant lines; *tzp pif4 pif5* 2.2.3, *tzp pif4 pif5* 1.20.4 are two independent *tzp pif4 pif5* triple mutant lines. Scale bar, 3 mm. Seedlings were scanned at the end of the fifth day. Hypocotyl length was measured from digital images using ImageJ. In whisker plots, boxes show median, IQR, and maximum–minimum interval of each data set (*n* = 15 seedlings). Data shown are representative of three biological replicates with independent populations of seedlings. Different lowercase letters represent significant differences by one-way ANOVA with Tukey’s post hoc test among assessed samples (*P* < 0.05).

As described above, phytochrome-mediated light signaling converges onto PIFs. PIF5 is partially redundant with PIF4 in regulation of hypocotyl elongation and both are required to fully promote hypocotyl growth (Lorrain et al., 2008). Studying the genetic interaction between TZP, PIF4, and PIF5 would help us clarify the position of TZP in red light signaling. For this purpose, we generated the double and higher-order mutant lines *tzp pif4* and *tzp pif4 pif5* and determined their hypocotyl elongation phenotypes. We observed that *pif4 pif5* double mutant seedlings exhibit shorter hypocotyl phenotypes than WT or *tzp*, thus exhibiting a hypersensitive response in red light (Figure 9), which suggests that PIF4 and PIF5 function as negative regulators in red light signaling. In red light and control dark conditions, *tzp pif4* resembled the *pif4* single mutant, while the triple *tzp pif4 pif5* mutant lines were indistinguishable from the *pif4 pif5* double mutant (Figure 9 and Supplemental Figure S9), indicating that PIF4 and PIF5 act downstream of TZP in the red light signaling pathway.

## Discussion

Since the initial identification of TZP by QTL mapping (Loudet et al., 2008), several studies have reported a function for TZP in blue light and far-red light-mediated photomorphogenesis and photoperiod regulation of flowering (Kaiserli et al., 2015; Perrella et al., 2018; Zhang et al., 2018; Li et al., 2021). The direct interaction between TZP and phyB in response to red light, as well as its association with clock and thermoregulatory components such as ELF3 (Kaiserli et al., 2015) and PCH1 (Huang et al., 2016b), indicates that TZP integrates multiple light, temperature, and photoperiod pathways. Yet, how TZP exerts its function and modulates signaling pathways in response to multiple environmental stimuli is still unclear. This study reveals that TZP integrates light and photoperiod signaling through a cooperative action with PCH1. Although the exact molecular mechanism of the relationship between TZP and PCH1 is not fully understood, our data uncovered their synergistic action in the control of hypocotyl elongation in LDs and red light. Furthermore, we showed that TZP acts as a positive regulator of phyB signaling by promoting phyB protein stability and NB formation.

### TZP and PCH1 play indispensable roles in red light signaling

Based on the experimental data described above (Figure 3E), we can conclude that TZP and PCH1 play synergistic roles in controlling phyB-mediated red light signaling. *pch1* mutants were previously reported to exhibit an elongated hypocotyl phenotype compared with WT in SDs due to an increase in PIF4 protein abundance in the early evening hours (Huang et al., 2016b). Under a LD photoperiod, phyB acts as the major suppressor of PIF4 and PCH1 function is potentially masked (Huang et al., 2016b), thus *pch1* mutants exhibit no obvious hypocotyl phenotypes under this condition. When associating with TZP, PCH1 function on phyB is exaggerated, thus the absence of the two proteins leads to impaired phyB function and PIF4 activity is maintained at a high level over the entire time course in LDs, which results in long hypocotyl phenotype in *tzp pch1* mutant lines. Furthermore, the long hypocotyl exhibited by *tzp pch1* is comparable to that of the null allele *phyB-9*, which indicates that in the absence of functional TZP and PCH1, seedlings display impaired sensitivity to light and in particular to red light (Figure 1). TZP and PCH1 therefore play indispensable roles in red light signaling. Collectively, we hypothesize that even though phyB maintains high protein abundance in *tzp pch1*, it is functionally impaired, probably due to the inability to form NBs. In addition, *OXTZP* partially rescued the elongated hypocotyl phenotype of *pch1* in red light and SDs (Figure 2), further supporting our conclusion that the attenuated response of *tzp pch1* to light is caused by the combined loss of TZP and PCH1.

To follow up, we studied the molecular mechanisms behind the impaired photomorphogenic phenotypes of *tzp pch1*, especially with regards to phyB-mediated signaling. As previously reported, phyB signaling converges on the regulation of PIF family members (Leivar and Quail, 2011). PCH1 was reported to influence *PIF4* transcript and PIF4 protein abundance in SDs (Huang et al., 2016b). Therefore, we specifically examined if PIF4 was regulated transcriptionally and post-transcriptionally and whether altered PIF4 levels might account for the *tzp pch1* phenotype. Our results showed that *tzp pch1* led to elevated PIF4 protein levels over a diurnal cycle in LDs, which suggests that even during the daytime (ZT0-16) when phyB is abundant, its effects on PIF4 degradation are largely attenuated in the absence of TZP and PCH1 (Figure 3E). These data indicate that TZP and PCH1 are essential for phyB-mediated light signaling.

Subsequently, we investigated the relationship between TZP and PCH1 at the cellular level. No direct interaction has been reported between TZP and PCH1 (Huang et al., 2016b), but our data show that the formation of TZP NBs is impaired in the absence of functional PCH1 (Figure 4B). More specifically, we detected fewer NBs in *pch1 OXTZP* (Figure 4), which was not surprising since TZP is known to require phyB for NB formation (Kaiserli et al., 2015) and PCH1 acts as a structural component of phyB NBs (Huang et al., 2019). Investigating a potential role for TZP in PCH1 localization and function would be very informative in deciphering the relationship between these two proteins.

### TZP is a positive regulator of phyB signaling

We further explored the role of TZP in modulating phyB function and discovered that TZP stabilizes phyB (Figure 6A), by potentially interfering with its proteasomal degradation (Figure 6E). Our data clearly show that TZP positively regulates phyB protein abundance, since the *tzp* mutant showed decreased phyB protein levels, whereas *OXTZP* led to increased phyB accumulation (Figure 6A). We observed a more dramatic difference between *OXPB* and *tzp OXPB*, where the lack of TZP led to a reduction in overexpressed phyB-CFP levels (Figure 6C). However, TZP-dependent regulation did not affect *PHYB* transcript levels, as *tzp OXPB* exhibited comparable *PHYB* expression to *OXPB* (Supplemental Figure S6D), indicating that TZP function on phyB is primarily at the protein level.

We further examined the molecular mechanism of TZP-dependent phyB protein accumulation. PhyB was reported to undergo red light-induced degradation mediated by the 26S proteasome (Jang et al., 2010). Thus, we tested whether TZP can protect phyB from proteasomal degradation. Our MG132 pharmacological applications showed that phyB degradation is intensified in the *tzp* mutant (Figure 6E). This study demonstrated that TZP can protect phyB from being degraded, although the mechanism by which TZP exerts this function remains unclear. TZP was reported to colocalize and interact with phyB (Kaiserli et al., 2015), therefore, one possibility could be that a direct interaction between TZP and phyB might interfere with COP1 binding, thus resulting in protecting phyB from COP1-mediated proteasomal degradation. How TZP affects ubiquitination of phyB by COP1 requires further investigation. Additionally, investigating a possible genetic interaction between TZP and COP1 in red light or whether TZP-dependent phyB stabilization modulates COP1 action would be of great interest, especially based on the recent discovery that TZP interacts with COP1 directly and that this interaction interferes with COP1 regulation on downstream components in far red light (Li et al., 2021). The effect of MG132 on phyB NB formation in *OXPB* and *tzp OXPB* using an identical treatment to the one applied for immunoblot analysis also showed an apparent decrease in the number of phyB NBs in *tzp OXPB* compared with *OXPB*. However, statistical analysis determined that this difference was not significant (Supplemental Figure S7B). Therefore, these results need to be interpreted with caution as the effect of MG132 in NB formation is not fully established. In addition to its role during inhibition of the proteasomal function, MG132 may also affect phyB NB formation by an unknown mechanism.

Together with the effect of TZP on phyB protein abundance, we observed a decrease in the number of phyB NBs in *tzp OXPB* lines compared with *OXPB* (Figure 7), however, we could not exclude the possibility that the impairment of NB formation in *tzp OXPB* is caused by the much lower phyB levels in this line (Figure 6C). We would need to uncouple the effect of TZP on phyB protein abundance to examine a potential regulatory role for TZP in phyB NB formation. To validate this hypothesis, we employed YHB, a constitutively active phyB variant, to explore this question (Su and Lagarias, 2007). We introgressed the *tzp* mutation in the *YHB* line and scored NB formation in *tzp YHB* and *YHB* in dark-grown seedlings (Figure 8). We detected large NBs in etiolated *YHB* seedlings; by contrast, we observed more and smaller NBs in *tzp YHB* (Figure 8, B and C), a phenotype similar to that reported for *hemera* (*hmr*) mutants (Chen et al., 2010). Therefore, TZP potentially affects the stability of phyB NBs regardless of total phyB protein abundance (Supplemental Figure S8). PCH1 is reported to act as a structural component for phyB NBs and YHB can hardly form NBs in *pch1*. Perhaps TZP has an accessory role in PCH1-mediated phyB NB formation or a role similar to HEMERA, which promotes phyB NB formation and PIF degradation (Chen et al., 2010). These data indicate that the effect of TZP on red light signaling is not only through the regulation on phyB protein abundance but also directly on the light signaling transduction, as NB formation closely relates to the regulation of downstream components and the ultimate phenotypic outcomes (Chen et al., 2003, 2010). Alternatively, the role of TZP on phyB NB formation could be examined in a heterologous system such as mammalian cells (Golonka et al., 2019). From this study on NB formation, we can also draw the conclusion that TZP showed weaker effects in red light signaling compared with PCH1. As in the study of PCH1, nearly no phyB NBs were detected in *pch1 YHB-YFP* (Huang et al., 2019). In comparison, even though TZP affects YHB NB formation, the influence was minor compared with PCH1, which may also explain why *pch1* displays insensitivity to red light whereas *tzp* shows no strong phenotype in red light.

Taken together, these data indicate that TZP regulates phyB and PIF4 levels in response to red light. Yet, the presence of a red-light-specific hypocotyl phenotype in *tzp pch1*, but not in the *tzp* single mutant remains puzzling, but it supports our hypothesis of TZP having a rather supporting role in this regard. Whether other phytochromes or downstream signaling components compensate for the decrease in phyB protein in *tzp* is a possible explanation that could be investigated in future studies. Therefore, the role of TZP in regulating PIF4 may be supported or exaggerated in the absence of functional PCH1. To be more specific, a lack of PCH1 may inhibit phyB physiological function additively with TZP and thus strengthen the effect of the *tzp* mutation, leading to impaired light responses and the long hypocotyl seen in the *tzp pch1* double mutant even in LDs (Figure 1A).

### PIF4 acts downstream of the TZP–phyB–PCH1 complex

PIF4 acts as the main integrator of different signaling pathways. phyB (Leivar et al., 2008), together with PCH1 (Huang et al., 2016b) has been shown to negatively regulate PIF4 activity by various mechanisms. In the study of the interaction between TZP and PIFs, we concluded that PIF4 and PIF5 act downstream of TZP (Figure 9). This work also demonstrated that TZP downregulates PIF4 protein levels, possibly by stabilizing phyB (Figure 6B). All pieces of evidence indicate that PIF4 acts downstream of each of the main proteins. Taking all this information into consideration, we can position PIF4 downstream of the TZP–phyB–PCH1 protein complex. In WT seedlings, activated phyB colocalizes with PIFs in NBs, triggering PIF phosphorylation and subsequent degradation (Lorrain et al., 2008; Dong et al., 2017). Decreased PIF4 levels lead to the inactivation of downstream growth-promoting genes, resulting in inhibition of hypocotyl growth (Kunihiro et al., 2011) and thus photomorphogenesis is facilitated. In the absence of TZP and PCH1, impaired phyB NB formation leads to higher PIF4 protein levels and increase in hypocotyl growth by activating the expression of downstream hypocotyl elongation marker genes. Overall, this study reveals novel roles for both TZP and PCH1 in fine-tuning photoperiodic hypocotyl growth by modulating phyB signaling. The role of TZP in regulating phyB signaling could be independent from or linked to PCH1. Further investigation of the localization and function of phyB in the *tzp pch1* double mutant and PCH1 in *tzp* would shed light on the molecular mechanism of this tripartite protein complex and clarify the relationship between TZP and PCH1.

Although there have been significant advances in our understanding of TZP function and role in bridging signaling networks in response to environmental stimuli, many unanswered questions remain: Does TZP regulate gene expression by controlling chromatin remodeling complexes? Are TZP and phyB NBs an example of biological condensates formed by liquid–liquid phase separation in response to light? Does TZP integrate light and temperature signaling? Understanding the mechanisms of environmental signal integration through TZP action will allow us to implement these findings in precision agricultural practices on economically important species to minimize crop loss and enhance food production in response to climate change.

## Materials and methods

### Plant material and growth conditions

The Arabidopsis accession Columbia-0 (Col-0) was used as WT unless otherwise indicated. The following mutants and transgenic lines used in this study were previously described: *tzp* (*tzp-1*) (SALK_069477.101), *35S:TZP-GFP* in Col-0 (*OXTZP*) (Kaiserli et al., 2015; Perrella et al., 2018), *pch1* (Nusinow Lab, Danforth Center, SALK_024229, [Huang et al., 2016b]), *phyB-9* (CS6217, [Reed et al., 1994]), *35S:PHYB-CFP* (*OXPB*) (Chory Lab, Salk Institute, [Nito et al., 2013]), *PHYB^Y276H^* (*YHB*) (Su and Lagarias, 2007), *pif4* (*pif4-101*) (SAIL_114_G06), and *pif5* (*pif5-3*) (SALK_087012) (Lorrain et al., 2008). Mutant combinations of *tzp* and *OXTZP* with *pch1* and *phyB-9* or *OXPB* that were generated were verified by genotyping; *pch1 35S*:*TZP-GFP* lines were generated by floral dipping; *pch1 OXTZP*, *tzp OXPB*, and *tzp YHB* were generated by crossing. Both Col-0 and the *tzp* mutation were introgressed in *YHB* (L*er*). Plants grown on soil were cultivated in growth room cabinets (Snidjers Photobiology Treatment Chamber, WEISS TECHNIK) with regulated temperature (22°C in the light, 18°C in the dark), 40% humidity, specific light intensity, and photoperiod as described in each experiment. The Aracon system (http://www.arasystem.com) was applied on individual transgenic plants to prevent genetic contamination. All soil substrates (Levington F2) used for plant growth were treated with insecticide (Calypso 0.08% v/v, Bayer) to prevent insect breeding. Specialized light treatments were conducted in growth chambers (Snidjers Micro Climate Series) under monochromatic red, blue, or far-red light, and various photoperiods (LDs, 16-h light/8-h dark) or SDs (8-h light/16-h dark). Visible light (400–700 nm) intensity was measured by a Li-250A light meter fitted with a sensor (Li-Cor). Far-red light (700–800 nm) intensity was measured by a Field Spectroradiometer (Apogee, USA) following the manufacturer’s instructions.

### Generation of transgenic lines and genotyping

Transgenic lines *pch1 35S:TZP-GFP* were generated by Agrobacterium (*A. tumefaciens*)-mediated transformation of *pch1* using a previously described construct (Kaiserli et al., 2015). Pelleted Agrobacterium cells harboring the *TZP-GFP* construct were resuspended in Arabidopsis floral dipping solution (5% [w/v] sucrose and 0.05% [v/v] Silwett-L77). The optical density was measured at 600 nm and adjusted to 0.8. Floral dipping was conducted twice within a 2-day interval. Homozygous transgenic lines were generated and selected as described previously (Kaiserli and Jenkins, 2007). T_3_ homozygous lines used for further experiments were validated based on segregation of the antibiotic resistance marker or by PCR-based genotyping according to the supplier’s recommendation. At least three independent transgenic lines were isolated and validated. Genotyping was performed on genomic DNA extracted according to Edwards et al. (1991) and using GoTaq DNA polymerase (Promega) according to the manufacturer’s instructions. All primers used for genotyping are listed in Supplemental Table S1.

### Hypocotyl measurements

Surface-sterilized (3-min incubation in 50% [v/v] sodium hypochlorite solution followed by three washes in double distilled sterile water) and stratified seeds were first exposed to white light (60 μmol m^−2^ s^−1^) for 4 h to synchronize and induce germination prior to exposure to the various light conditions described in the figure legends before hypocotyl measurements were recorded. Seedlings were scanned on the end of the fifth day post-germination. Hypocotyl length was measured from digital images using the ImageJ software (Schindelin et al., 2012). An average of 15 seedlings was measured for each treatment described. Data were plotted and statistical analyses were performed using Excel, SPSS Statistics, and GraphPad Prism. In whisker plots, boxes show median, interquartile range (IQR), and maximum–minimum interval of each dataset together with individual data points. Data shown are representative of three biological replicates with independent populations of seedlings exposed to an identical treatment. A summary of the statistical analysis is shown in Supplemental Data Set S1.

### RNA extraction and gene expression analysis

Total RNA was extracted from approximately 80 mg of 4-day-old light grown pooled seedling tissue (or as indicated in the figure legend) with an RNeasy plant mini kit (Qiagen). First-strand cDNA was obtained from 1 µg total RNA using a Quantitect reverse transcription kit (Qiagen) following the manufacturer’s instructions. qPCR was performed with Brilliant III Ultra-Fast SYBR QPCR Master Mix (Agilent Technologies) on a StepOnePlus Real-Time PCR system (Applied Biosystems, Life Technologies). All primers used for qPCR are listed in Supplemental Table S2). Cycling conditions were performed as described previously (Perrella et al., 2018). Data analysis was conducted by implementing the Δ–ΔCT method for calculating relative expression. The expression of each gene of interest was first normalized to the housekeeping gene *IRON SULFUR CLUSTER ASSEMBLY 1* (*ISU1*), followed by normalizing against the WT (Col-0).

### Protein isolation and immunoblot analysis

Approximately 100 mg of 4-day-old light-grown pooled seedling tissue (or as indicated in the figure legend) was used for total protein isolation by directly grinding the samples in 1.5 volumes of 4× Laemmli buffer (250 mM Tris–HCl pH 6.8, 10% [w/v] SDS, 20% [v/v] β-mercaptoethanol, 40% [v/v] glycerol, and 0.1% [w/v] bromophenol blue). After vortexing, the mixture was boiled at 100°C for 5 min prior to centrifugation for 1 min at room temperature (22°C) at 15,700 × *g*. The supernatant was used for SDS-PAGE analysis. Equal volumes of total protein were separated in 4%–12% SDS-PAGE Bolt gels (ThermoFisher) followed by transfer and immunodetection using specific antibodies (details are given in figure legends) as previously described (Kaiserli et al., 2015). Primary antibodies used in this study were anti-phyB (1:3,000 [v/v], from Prof. Akira Nagatani Lab), anti-PIF4 (1:1,000 [v/v], Agrisera AS163955), anti-GFP (1:3,000 [v/v], Roche 11814460001), and anti-UGPase (1:10,000 [v/v], Agrisera AS05086). MG132 treatments were performed as described in the figure legend following previously described assays (Jang et al., 2010). Protein levels were quantified by band intensities with ImageJ.

### Confocal imaging and analysis

Seedlings from each treatment were mounted onto slides containing a droplet of dH_2_O (without fixation) and were imaged under a Leica SP8 confocal microscope. Cells from the outermost epidermis of the upper third of the hypocotyl in each line were monitored. GFP (TZP–GFP) fluorescence was detected using a 488-nm excitation laser and fluorescence emission was collected between 500 and 540 nm. CFP (phyB-CFP) fluorescence was monitored using excitation at 405 nm and emission between 460 and 505 nm. PhyB^Y276H^ was detected using excitation at 633 nm and emission at 650 nm. NBs were imaged with a 40× or 63× oil-immersion lens under tunable excitation from 470 to 670 nm. Images were acquired with a line average of 16. The number of NBs per nucleus or NB area was quantified and analyzed using ImageJ and data were plotted in GraphPad Prism. In whisker plots, boxes show median, IQR, and maximum–minimum interval of each data set. IQR was calculated by the formula: IQR = quartile_3_ (*Q*_3_) – quartile_1_ (*Q*_1_). Whiskers were obtained with the formula *Q*_1_ – 1.5 × IQR and *Q*_3_ + 1.5 × IQR. A minimum of 12 nuclei per condition and per biological replicate were examined. Asterisks (***) represent *P *<* *0.001 by one-way analysis of variance (ANOVA) and indicate significant differences between the compared lines. Representative images and data from three biological replicates with independent populations of seedlings are shown in this study. A summary of the statistical analysis is shown in Supplemental Data Set S1.

## Accession numbers

*TZP* (At5g43630), *PHYB* (At2g18790), *PIF4* (At2g43010), *PCH1* (AT2G16365), *ATHB2* (AT4G16780), *HFR1* (AT1G02340), *UGP* (AT3G03250), and *ISU1* (AT4G22220).

## Supplemental data

The following materials are available in the online version of this article.

**Supplemental Figure S1.** Synergistic interaction between TZP and PCH1 in far-red light.

**Supplemental Figure S2.** PCH1 is required by OXTZP-mediated hypocotyl elongation in LD, blue light, and far-red light.

**Supplemental Figure S3.** PIF4 protein and *PIF4* transcript levels in *tzp pch1*.

**Supplemental Figure S4.** PCH1 modulates TZP nuclear body formation.

**Supplemental Figure S5.** PhyB plays a role in blue light working associatively with TZP.

**Supplemental Figure S6.** TZP controls phyB protein abundance.

**Supplemental Figure S7.** TZP modulates phyB nuclear body formation.

**Supplemental Figure S8.** Effect of TZP on the protein abundance of phyB^Y276H^.

**Supplemental Figure S9.** PIF4 acts downstream of TZP in red light signaling.

**Supplemental Table S1.** Primers used in this study.

**Supplemental Table S2.** Primers used for qPCR.

**Supplemental Data Set S1.** Summary of statistical analyses.

## Supplementary Material

koac236_Supplementary_Data

## Notes

W.F., E.V., E.K., and G.P. designed, performed, analyzed, and interpreted the experiments. W.F. and G.P. carried out genetic crosses, genotyping, and generation of transgenic lines. W.F. and E.V. performed the hypocotyl assays and western blot analysis. W.F. executed all RT-qPCR studies. W.F. and E.K. performed the confocal image analysis. W.F., G.P., E.V., and E.K. wrote the article.

The author responsible for distribution of materials integral to the findings presented in this article in accordance with the policy described in the Instructions for Authors (https://academic.oup.com/plcell) is: Eirini Kaiserli (eirini.kaiserli@glasgow.ac.uk).
