# Supplementary material for: TANDEM ZINC-FINGER/PLUS3 regulates phytochrome B abundance and signaling to fine-tune hypocotyl growth
Source: Plant Cell. 2022 Aug 5;34(11):4213–31. doi: 10.1093/plcell/koac236 (PMC9614508; doi:10.1093/plcell/koac236)
Supplement: koac236_Supplementary_Data [file koac236_supplementary_data.zip › koac236_Supplementary_Data/TPC2022-RA-00278DR1 Suppl Figures revised.pdf]

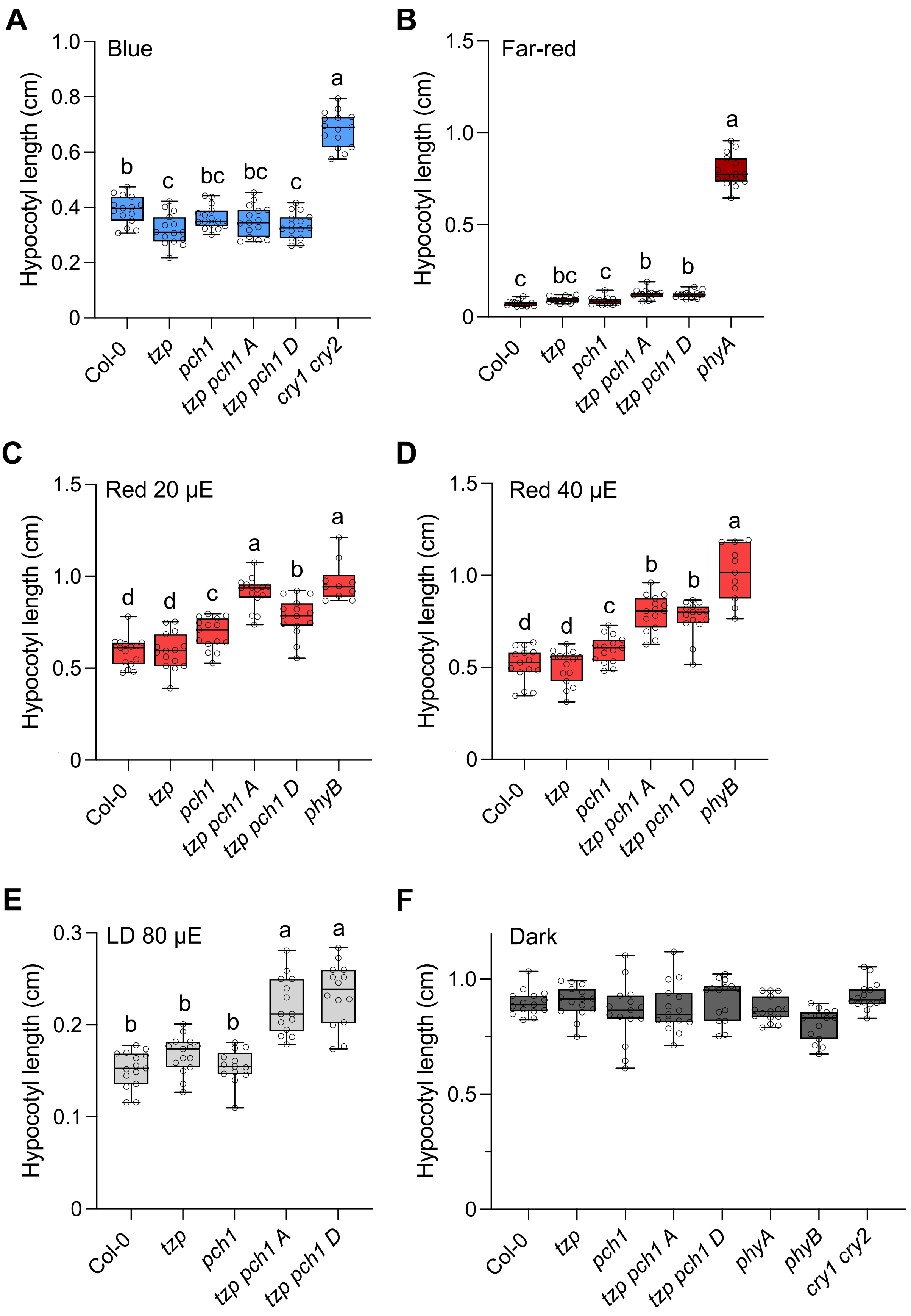

Supplemental Figure S1. Synergistic interaction between TZP and PCH1 in far-red light. See next page for full legend.

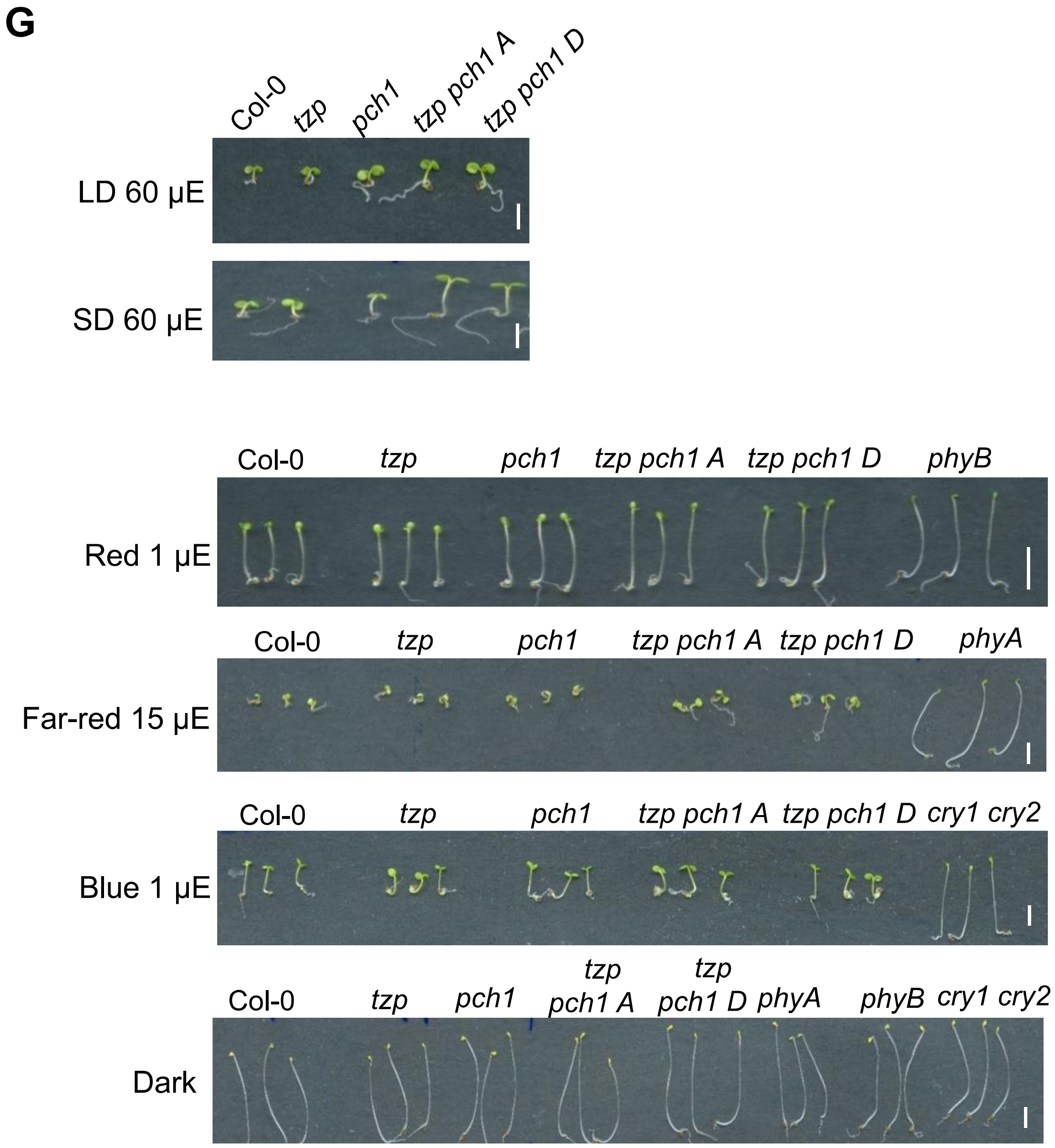

Supplemental Figure S1 (continued). Synergistic interaction between TZP and PCH1 in far-red light. See next page for full legend.

H

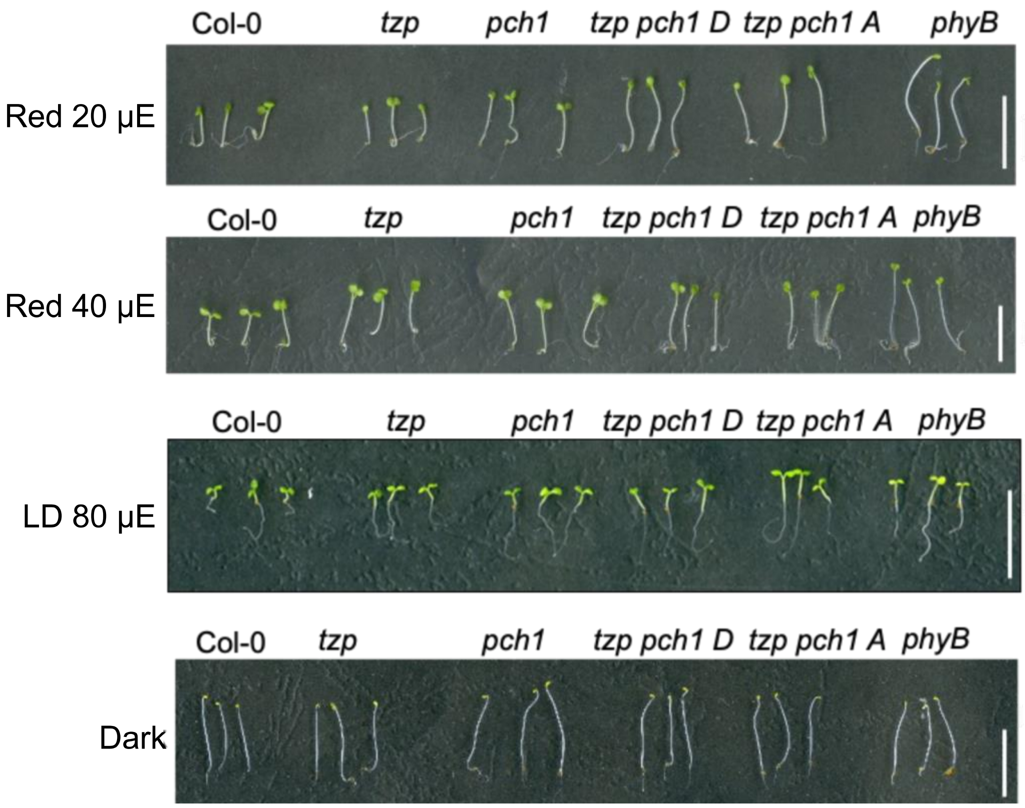

Supplemental Figure S1 (continued). Synergistic interaction between TZP and PCH1 in far-red light.

(A-F) Hypocotyl measurements of mutant combinations between *tzp* and *pch1* and controls. Seedlings were grown for 5 d in blue light (1 µmol m^{-2} s^{-1}) (**A**), far-red light (15 µmol m^{-2} s^{-1}) (**B**), red light (20 µmol m^{-2} s^{-1}) (**C**), red light (40 µmol m^{-2} s^{-1}) (**D**), white light (80 µmol m^{-2} s^{-1}) in long-day conditions (**E**) or darkness (**F**). Seedlings were scanned in the end of the fifth day. Hypocotyl lengths were measured from digital images using ImageJ. In whisker plots, boxes show median, interquartile range (IQR) and maximum-minimum interval of each data set (n=15 seedlings). Interquartile range was calculated with the formula: IQR=$quartile_3$ (Q_3) – $quartile_1$ (Q_1). Whiskers were calculated by Q_1 –1.5 × IQR and Q_3 + 1.5 × IQR. Different lowercase letters represent significant differences by one-way ANOVA with Tukey's post hoc test between assessed samples (P<0.05). Data shown are representative of three biological replicates with independent populations of seedlings. (G-H) Representative images of Col-0, *tzp*, *pch1, tzp pch1 A, tzp pch1 D, phyA, phyB, cry1 cry2* seedlings grown in LDs, SDs, red, blue, far-red light and darkness as indicated in Figure 1 and Supplemental Figure 1A-F. Scale bar, 2 mm (G) or 1 cm (H). Light fluence rate units: µE = µmol m^{-2} s^{-1}. LD = long day, SD = short day.
(supports Figure 1).

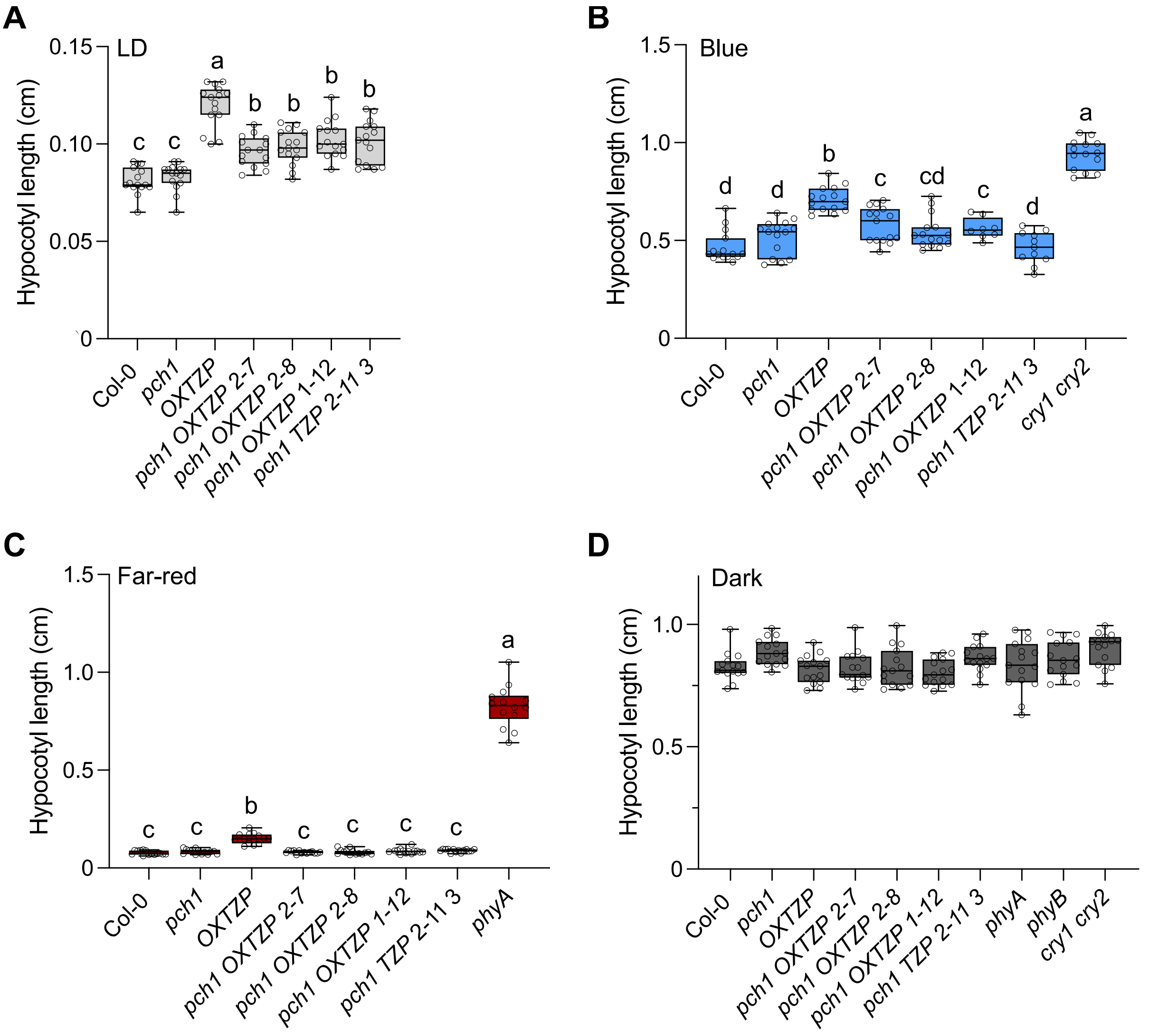

Supplemental Figure S2. **PCH1 is required by OXTZP mediated hypocotyl elongation in LD, blue light and far-red light.** See next page for full legend.

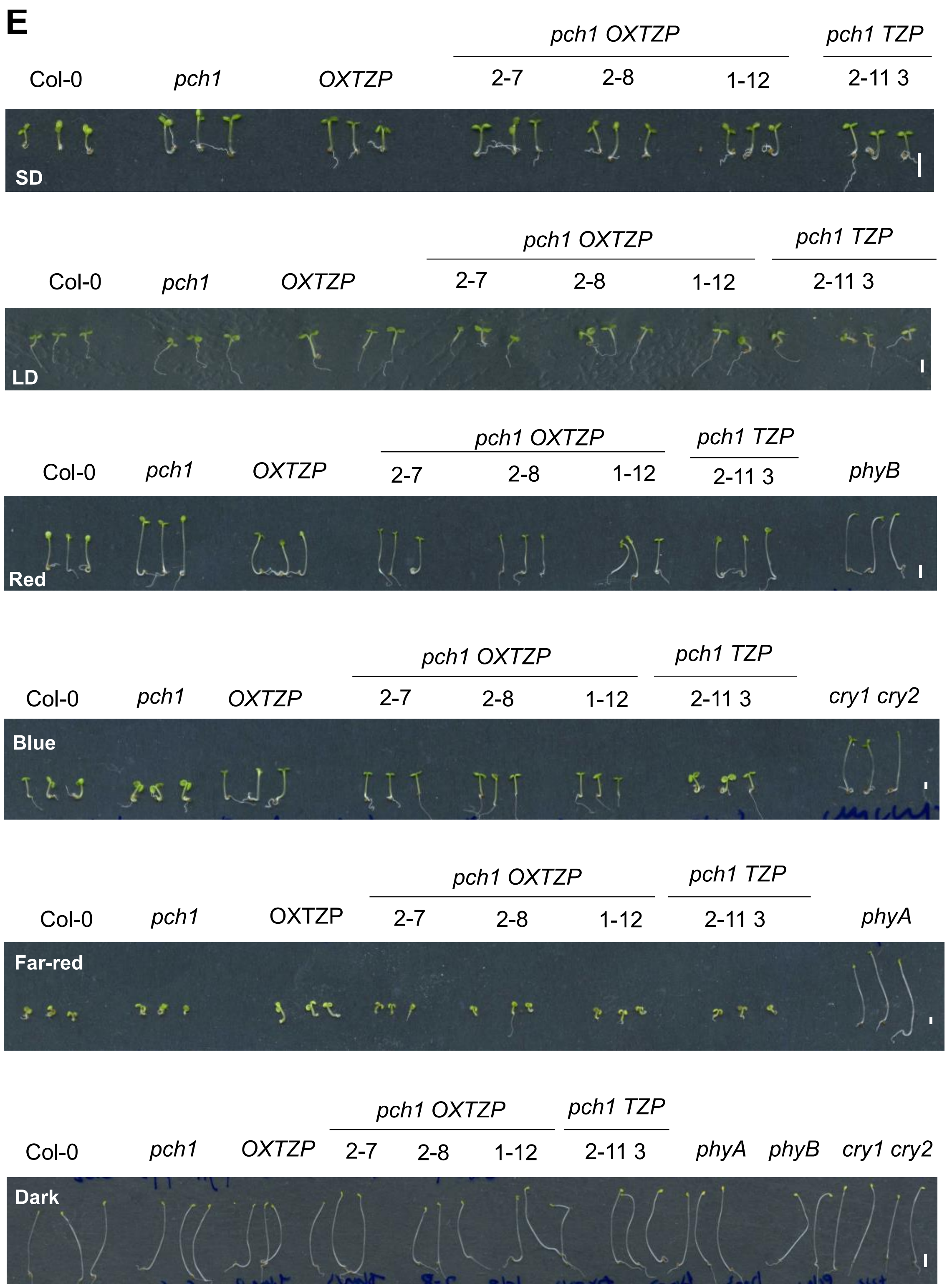

Supplemental Figure S2 (continued). PCH1 is required by OXTZP mediated hypocotyl elongation in LD, blue light and far-red light. See next page for full legend.

F

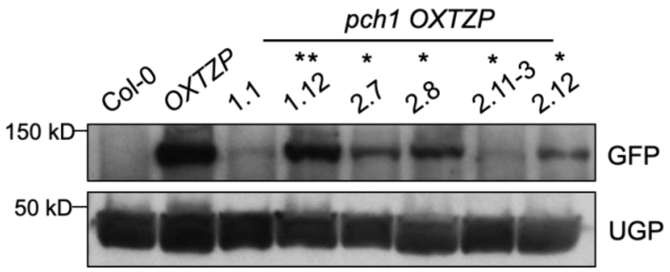

G

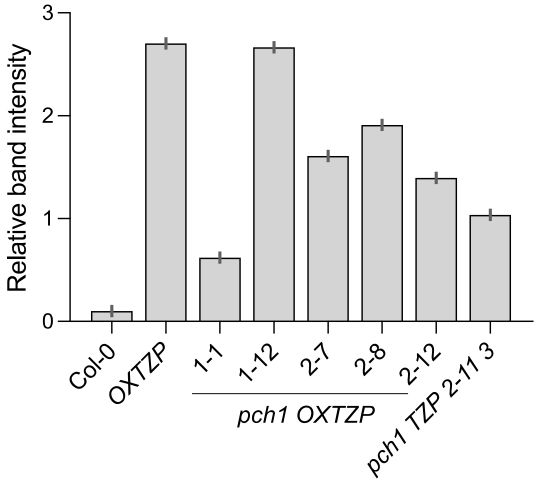

H

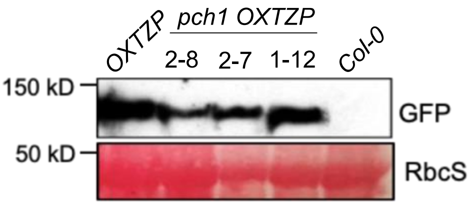

I

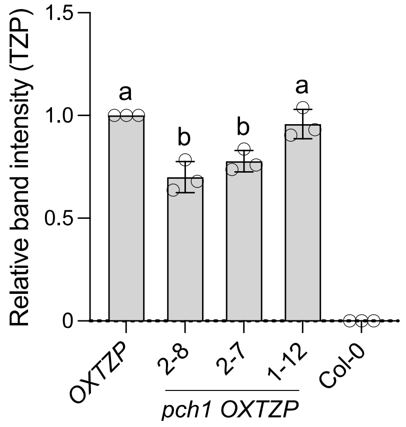

Supplemental Figure S2 (continued). PCH1 is required by OXTZP mediated hypocotyl elongation in LD, blue light and far-red light.
(A-D) Hypocotyl measurements of Col-0, *OXTZP*, *pch1*, genetic crosses of *pch1 OXTZP* (2-7, 2-8, 1-12), transgenic line *pch1 TZP* (2-11 3) and controls (*phyB, phyA, cry1 cry2*). Seedlings were grown for 5 d in white light (60 µmol m^{-2} s^{-1}) under a long day (16-h light/8-h dark) photoperiod (**A**), blue light (1 µmol m^{-2} s^{-1}) (**B**), far-red light (15 µmol m^{-2} s^{-1}) (**C**) or darkness (**D**). In whisker plots, boxes show median, interquartile range (IQR) and maximum-minimum interval of each data set (n=15 seedlings). Different lowercase letters represent significant differences by one-way ANOVA with Tukey's post hoc test among assessed samples (P<0.05). Data shown are representative of three biological replicates with independent populations of seedlings. **(E)** Representative images of seedlings used for hypocotyl measurements of Col-0, *pch1, OXTZP*, three crosses of *pch1 OXTZP* lines (2-7, 2-8, 1-12), one transgenic line *pch1 TZP* (2-11 3) with photoreceptor mutant *phyA, phyB, cry1 cry2* seedlings grown in conditions as indicated in Figure 2A-B and Supplemental Figure S2A-D. Scale bar, 1 mm. **(F)**

Immunoblot analysis of total protein extracts from white light (60 µmol m^{-2} s^{-1})-grown GFP-tagged *OXTZP* and *pch1 OXTZP* genetic crosses (2-7, 2-8, 1-12), and transgenic line (2-11 3) used for hypocotyl measurements and confocal imaging. An anti-GFP antibody was used to detect TZP and anti-UGP was used as a loading control. An asterisk (*) indicates the lines selected for hypocotyl measurements and (**) for quantitative confocal imaging. *OXTZP* was used as positive control; Col-0 was used as negative control. **(G)** Quantification analysis of (F) based on the relative immunoblot band intensities (GFP/UGP) using ImageJ. **(H)** TZP-GFP protein levels in *OXTZP* and *pch1 OXTZP* lines selected for confocal imaging from 7-d-old white light-grown seedlings (60 µmol m^{-2} s^{-1}). TZP protein levels were analyzed by immunoblot analysis using an anti GFP-specific antibody. A ponceau S staining of RbcS was used as loading control. Col-0 was used as negative control. An asterisk (*) indicates the line used for NB formation in comparison with OXTZP. **(I)** Quantification of relative band intensity (GFP/RbcS) of (H) was analyzed in ImageJ.
(supports Figure 2).

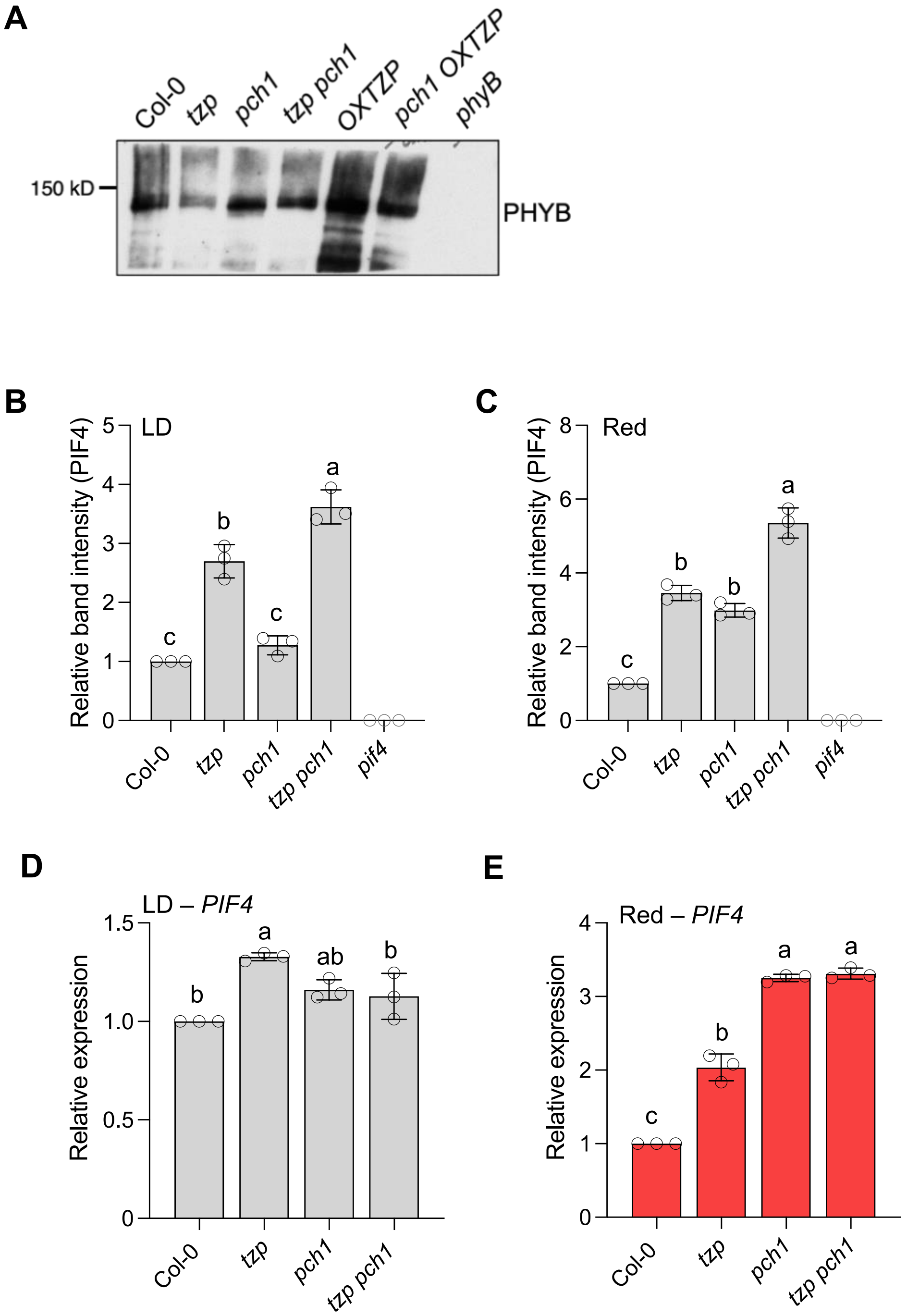

Supplemental Figure S3. PIF4 protein and *PIF4* transcript levels in *tzp pch1*.
(A) Full image of the immunoblot shown in Figure 3A. **(B-C)** Quantification of relative band intensities (PIF4/UGP) from the immunoblot analysis shown in Figure 3C and 3D, respectively. Data are shown as mean ± SD from three biological replicates with independent pools of tissue. Different lowercase letters represent significant differences by one-way ANOVA with Tukey's post hoc test (P<0.05). **(D-E)** RT-qPCR analysis of *PIF4* in WT, *tzp, pch1* and *tzp pch1*. Surface sterilized and stratified seeds were exposed to white light (60 μmol m^{-2} s^{-1}) under a LD photoperiod (**D**) or constant red light (1 μmol m^{-2} s^{-1}) (**E**) for 4 d. Tissue was collected on the fifth day at ZT8 for LD. Error bars represent SE of three biological replicates with independent pools of tissue. Different lowercase letters represent significant differences by one-way ANOVA with Tukey's post hoc test (P<0.05).
(supports Figure 3).

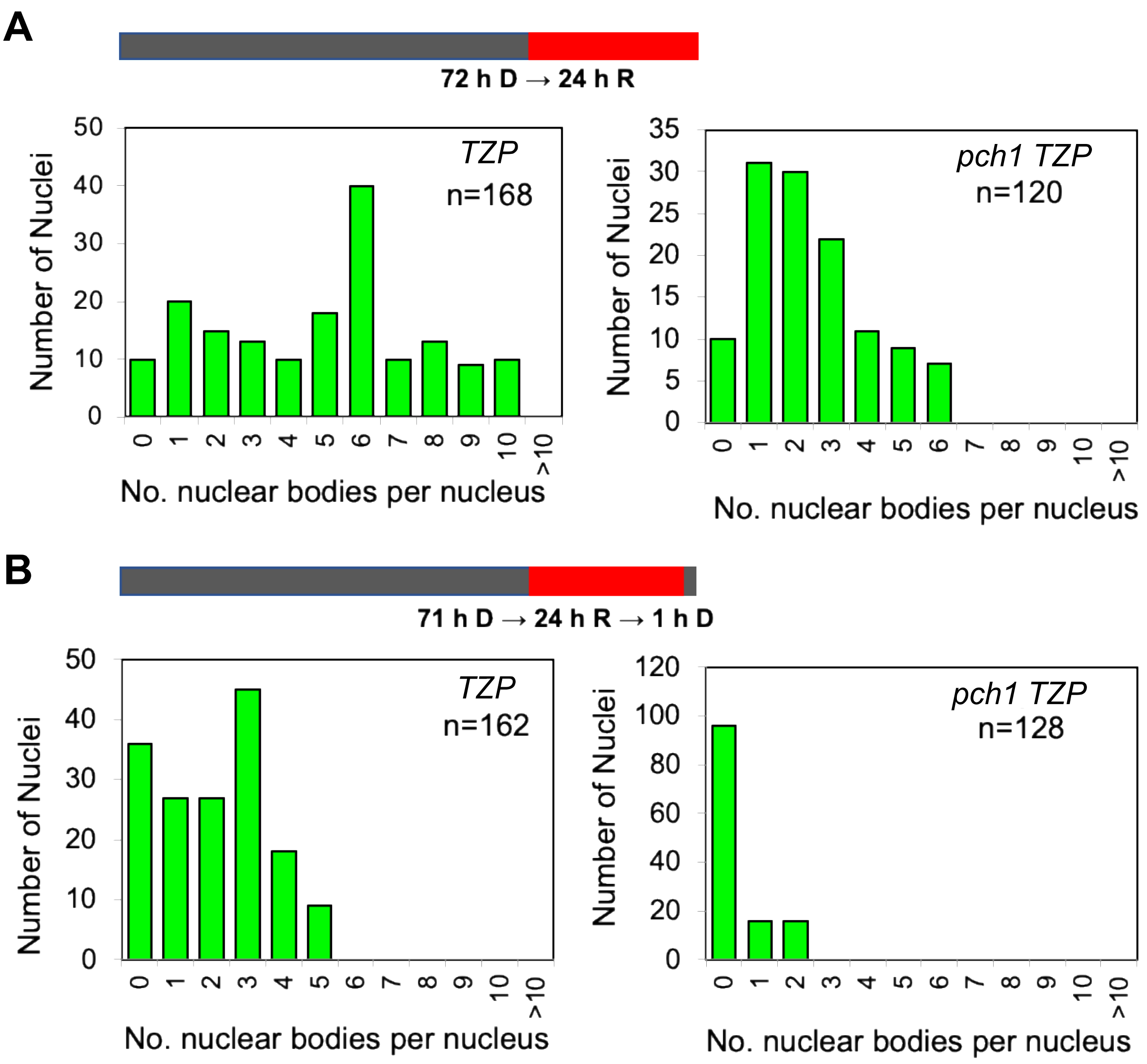

Supplemental Figure S4. PCH1 modulates TZP nuclear body formation.
Nuclear body formation and disassociation in *TZP* and *pch1 TZP* (line 1-12) monitored by confocal microscopy. Quantification of the confocal image analysis showing the number of TZP nuclear bodies per nucleus in *TZP* and *pch1 TZP* epidermis of the upper third part of hypocotyls in each treatment indicated. Four-day-old etiolated seedlings of *TZP* and *pch1 TZP* were exposed to: **(A)** red light (20 μmol m^{-2} s^{-1}) for 24 h; **(B)** red light (20 μmol m^{-2} s^{-1}) for 24 h followed by 1 h in the dark. "n" indicates the number of total nuclei examined. Confocal images were analyzed in ImageJ. Data shown are representative of three biological replicates with independent populations of seedlings.
(supports Figure 4)

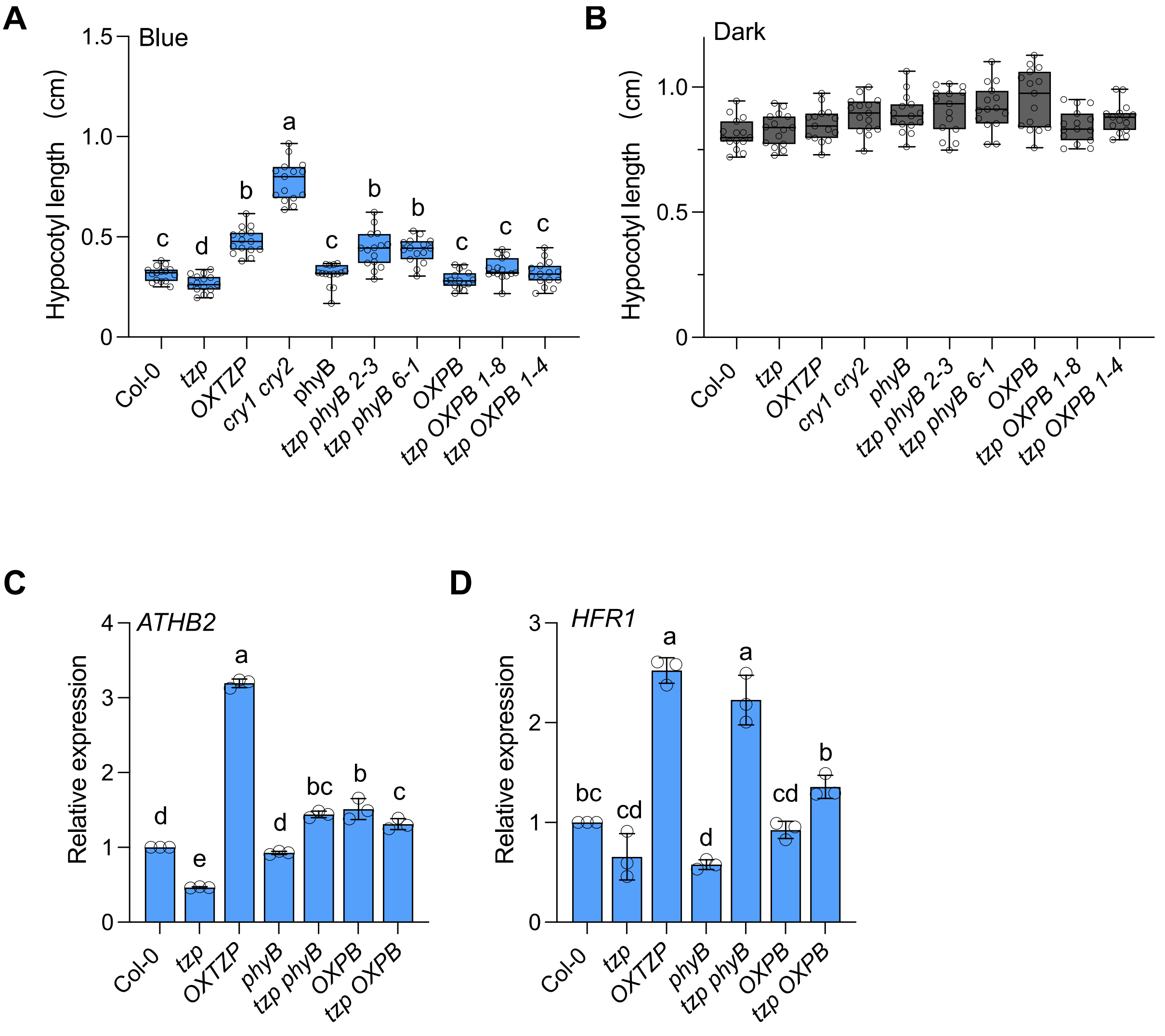

Supplemental Figure S5. PhyB plays a role in blue light working associatively with TZP.
(A-B) Hypocotyl measurements of mutant combinations between *tzp* and *phyB* or *OXPB* and the corresponding parental lines. Surface sterilized and stratified seeds were grown for 5 d in blue light (1 μmol m^{-2} s^{-1}) (**A**) or kept in darkness (**B**). Seedlings were scanned in the end of the fifth day. Hypocotyl length was measured from digital images using ImageJ. In whisker plots, boxes show median, interquartile range (IQR) and maximum-minimum interval of each data set (n=15 seedlings). Different lowercase letters represent significant differences by one-way ANOVA with Tukey's post hoc test among assessed samples ($P<0.05$). Data shown are representative of three biological replicates with independent populations of seedlings. **(C-D)** RT-qPCR analysis of *ATHB2* (**C**) and *HFR1* (**D**) mRNA levels normalized to the housekeeping gene *ISU1* of the indicated genotypes**.** Surface sterilized and stratified seeds were exposed to 5 μmol m^{-2} s^{-1} blue light; seedlings were harvested on the fifth day after being exposed to light. Error bars represent SE of three biological replicates with independent pools of tissue. Different lowercase letters represent significant differences by one-way ANOVA with Tukey's post hoc test ($P<0.05$).
(supports Figure 5).

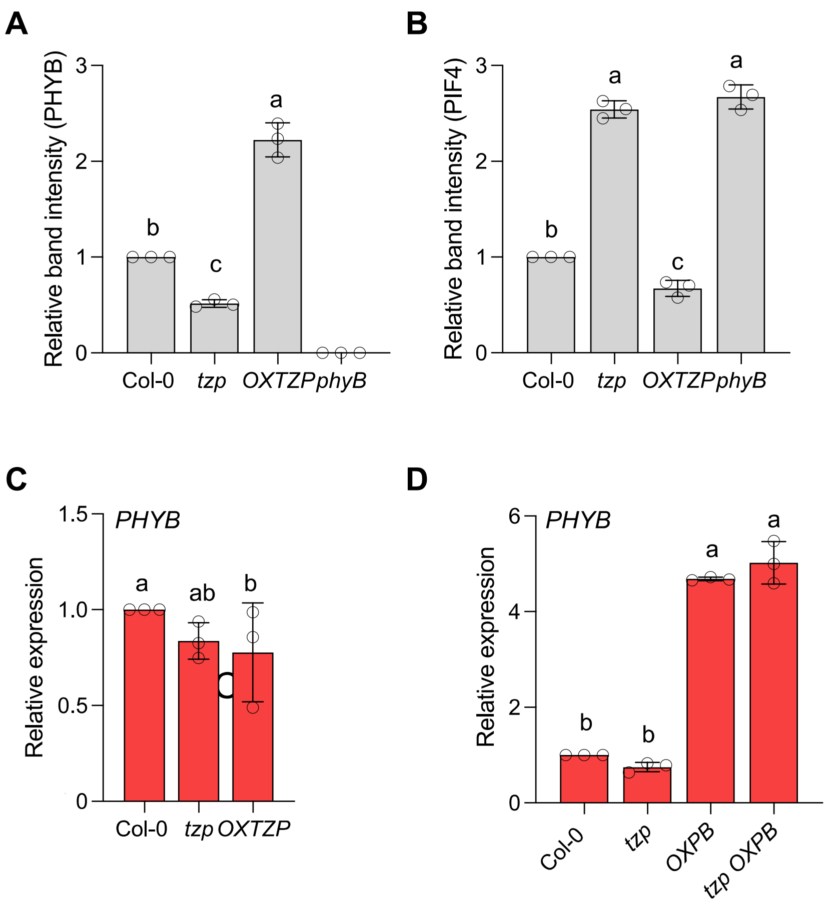

Supplemental Figure S6. TZP controls phyB protein. See next page for full legend.

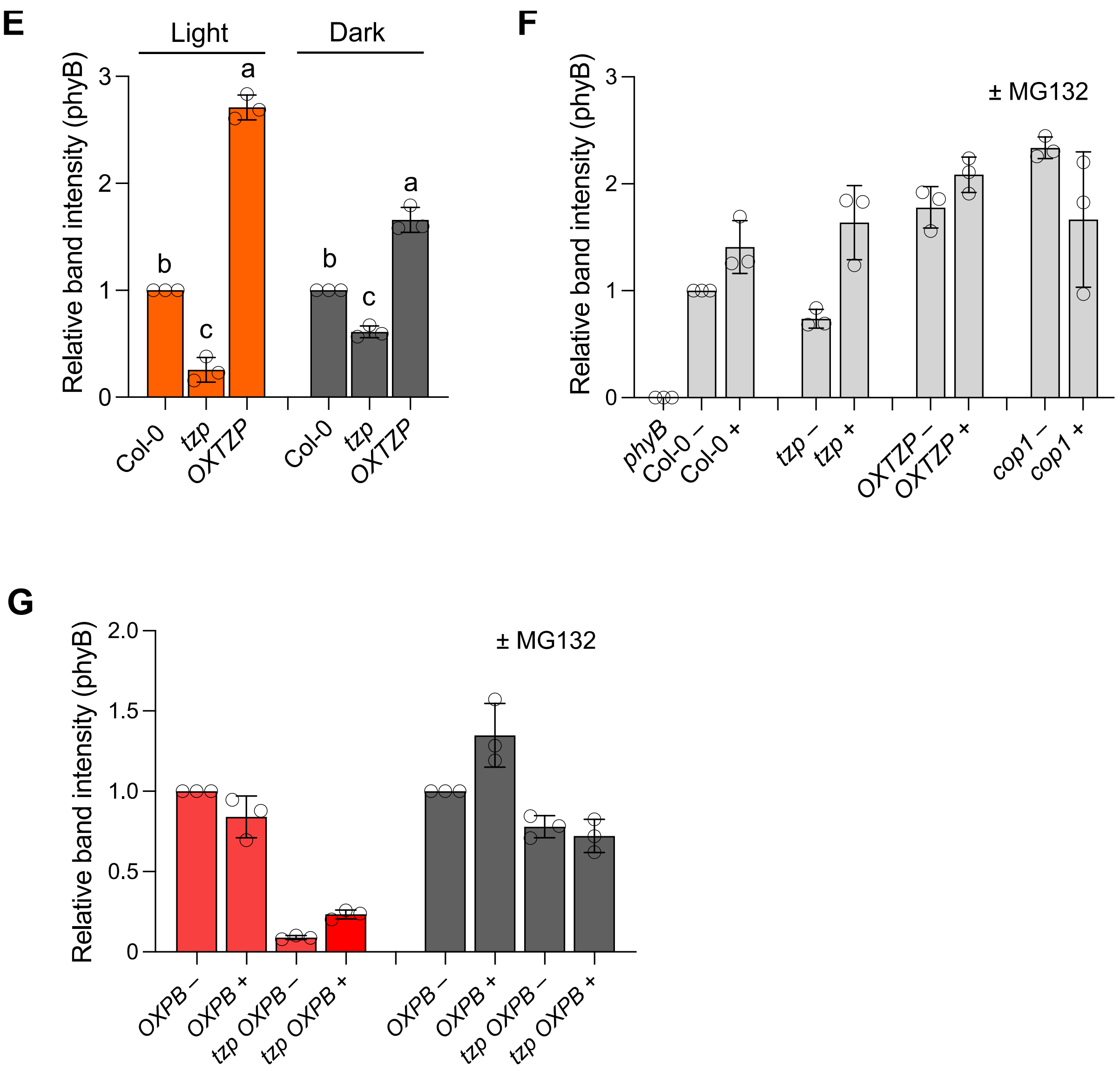

Supplemental Figure S6 (continued). TZP controls phyB protein abundance.
(A) Quantification of relative band intensities in Figure 6A from seedlings grown in constant red light (1 μmol m^{-2} s^{-1}) for 4 d using an anti-phyB-specific antibody. **(B)** Quantification of relative band intensities of Figure 6B immunoblot analysis of PIF4 protein levels of seedlings grown under the same condition as Figure 6A. **(C-D)** RT-qPCR analysis of *PHYB* mRNA levels normalized to the housekeeping gene *ISU1* of the indicated genotypes in seedlings exposed to 1 μmol m^{-2} s^{-1} red light for 4 d. Error bars represent SE of three biological replicates with independent pools of tissue. Different lowercase letters represent significant differences by one-way ANOVA with Tukey's post hoc test (P<0.05). **(E)** Quantification of relative band intensities (PHYB/UGP) of the immunoblot shown in Figure 6D. Error bars represent SE of three biological replicates with independent pools of tissue. Different lowercase letters represent significant differences by one-way ANOVA with Tukey's post hoc test (P<0.05). **(F)** Quantification of relative band intensities (PHYB/UGP) of the immunoblot shown in Figure 6E. Error bars represent SE of three biological replicates with independent pools of tissue. **(G)** Quantification of the relative band intensities (PHYB/UGP) of the immunoblot shown in Figure 6F. Error bars represent SE of three biological replicates with independent pools of tissue.
(supports Figure 6).

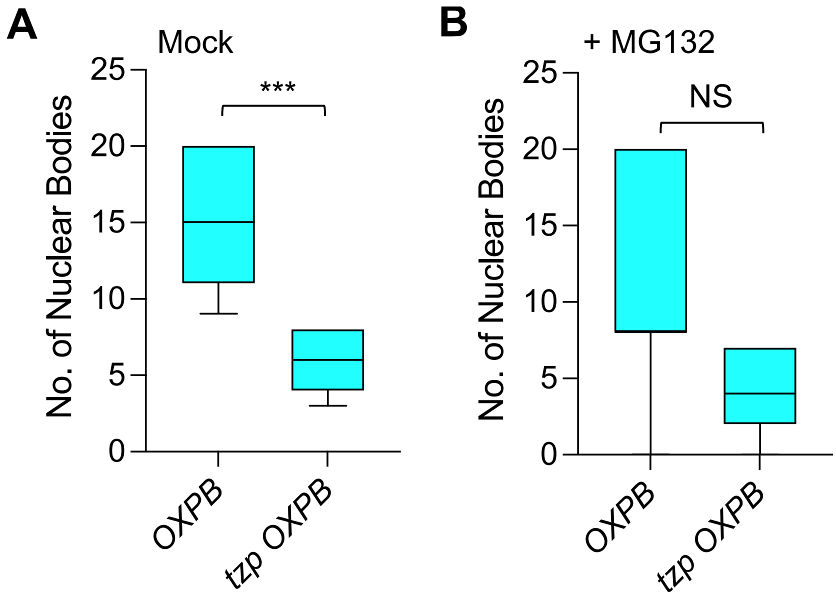

Supplemental Figure S7. TZP modulates phyB nuclear body formation.
Confocal image analysis of 4-d-old etiolated seedlings expressing *OXPB* or *tzp OXPB* infiltrated with 50 μM of the proteasomal inhibitor MG132 or an equivalent volume of DMSO in liquid half-strength MS medium for 2 h in the dark prior to a 2-h red light (25 μmol m^{-2} s^{-1}) exposure as described in Figure 6E. **(A-B)** Quantification analysis of the number of nuclear bodies per nucleus from confocal images of *OXPB* and *tzp OXPB* in response to a mock (**A**) or MG132 (**B**) treatment. Confocal images were analyzed in ImageJ and graphs were plotted in GraphPad Prism. In whisker plots, boxes show median, interquartile range (IQR) and maximum-minimum interval of each data set. Asterisks (***) represent *P*<0.001 by one-way ANOVA and indicate significant difference compared to *OXPB*. “NS” indicates non-significant difference. A minimum of 10 nuclei per treatment were examined. Data shown are representative of three biological replicates with independent populations of seedlings.
(supports Figure 7).

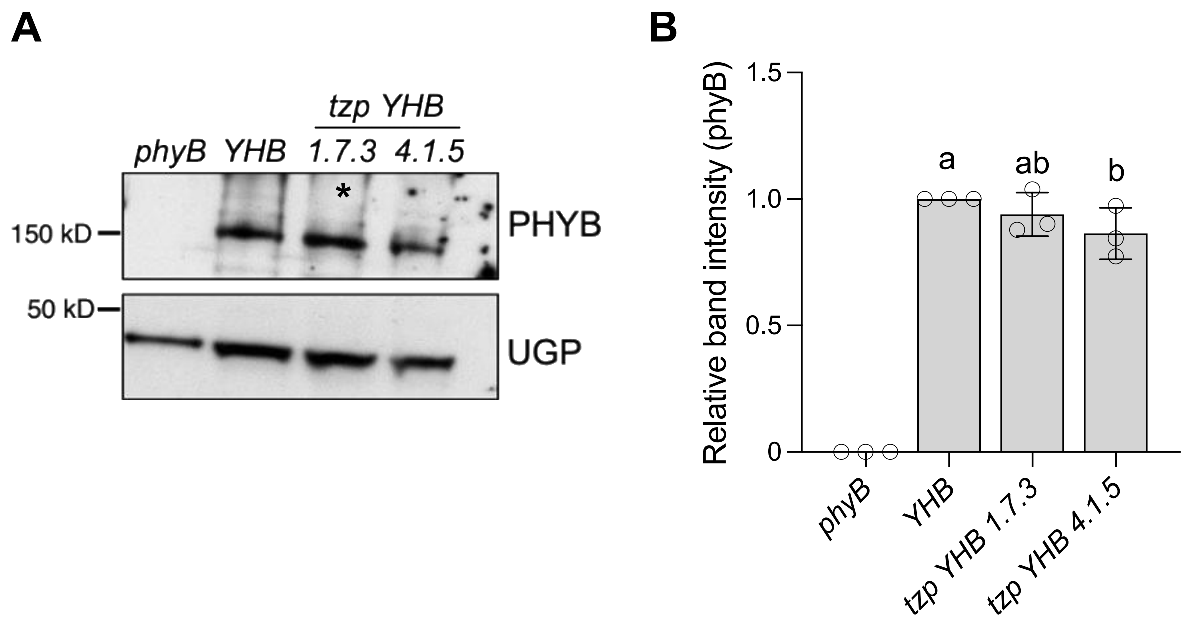

Supplemental Figure S8. Effect of TZP on the protein abundance of phyB^{Y276H}.
(A) Immunoblot analysis of phyB levels in *YHB* and *tzp YHB*. Seedlings were prepared as described in Figure 8A. Four-day-old dark-grown *YHB* and *tzp YHB* seedlings were harvested for protein extraction. phyB protein levels were analyzed with an anti-phyB-specific antibody, UGP was used as loading control. An asterisk (*) indicates the line selected for quantitative confocal imaging used in Figure 8. **(B)** Quantification of relative band intensities (PHYB /UGP) of the immunoblot shown in (A). Error bars represent SE of three biological replicates with independent pools of tissue. Different lowercase letters represent significant differences by one-way ANOVA with Duncan's post hoc test ($P<0.05$).
(supports Figure 8).

A

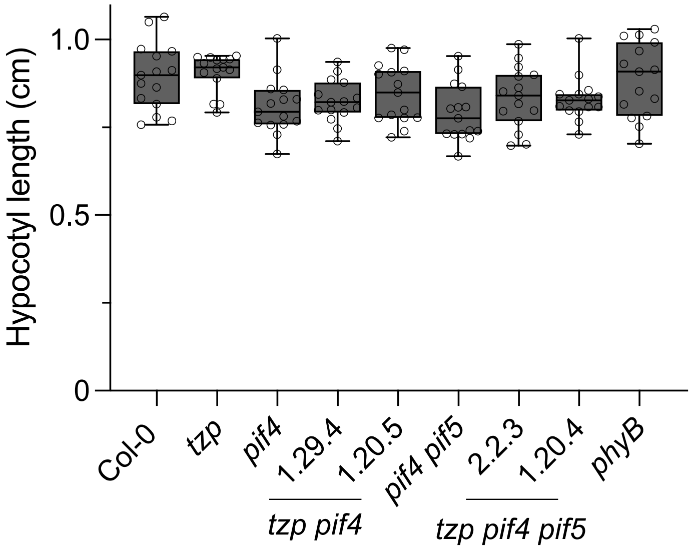

B

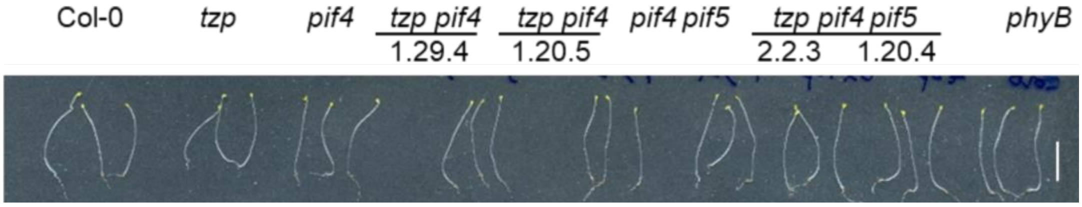

Supplemental Figure S9. PIF4 acts downstream of TZP in red light signaling.
Hypocotyl measurements of mutant combinations between *tzp* with *pif4, pif4 pif5* and controls. Seedlings were grown for 5 d in darkness. Lengths of measured hypocotyl (**A**) and representative images (**B**) of Col-0, *tzp*, *pif4*, *tzp pif4* 1.29.4, *tzp pif4* 1.20.5*, pif4 pif5, tzp pif4 pif5 2.2.3*, *tzp pif4 pif5 1.20.4, phyB* seedlings grown in the indicated conditions. Scale bar, 3 mm. *tzp pif4* 1.29.4, *tzp pif4* 1.20.5 are two independent *tzp pif4* double mutant lines; *tzp pif4 pif5 2.2.3*, *tzp pif4 pif5 1.20.4* are two independent *tzp pif4 pif5* triple mutant lines. Seedlings were scanned at the end of the fifth day. Hypocotyl length was measured from digital images using ImageJ. In whisker plots, boxes show median, interquartile range (IQR) and maximum-minimum interval of each data set (n=15 seedlings). Data shown are representative of three biological replicates with independent populations of seedlings. **(supports Figure 9)**

Supplemental Table S1. Primers used in this study.

For genotyping		
	For wild type PCR (5'-3')	For mutant PCR (5'-3')
tzp	ACGCTTCTTCTCTTCCATTCC (LP)<br>TGTTTGGGGTCAACTTCAAAG (RP)	TGTTTGGGGTCAACTTCAAAG (RP)<br>ATTTTGCCGATTTCGGAAC (LBb 1.3)
pch1	TGTCAGGTATTTCGGTCCTTG (LP)<br>CACTTGCTTGATGCTCATGAG (RP)	AAGAACCGGCAAAGATACCAC (RP)<br>ATTTTGCCGATTTCGGAAC (LBb 1.3)
pif4	AATACATTTTGCAGGCAATCG (LP)<br>CGTAATGAAGTTGCACGTTTACTC (RP)	CGTAATGAAGTTGCACGTTTACTC (RP)<br>TAGCATCTGAATTTCATAACCAATCTCGATAAC (LB3)
pif5	CGATTTGTTACCCATGCTTTG (LP)<br>CCTTGCTGGATTTTTGTTACG (RP)	CCTTGCTGGATTTTTGTTACG (RP)<br>ATTTTGCCGATTTCGGAAC (LBb 1.3)

For qPCR		
	Forward primers (5'-3')	Reverse primers (5'-3')
HFR1	GATGCCATCGCCGCTAATT	GTAAACGGTGGTAATGGCCAAT
ATHB2	ACCATGTGCCCTTCATGTGA	CTGACGTAGCAGCCTGAGGTT
PIF4	GTTGTTGACTTTGCTGTCCCGC	CCAGATCATCTCCGACCGGTTT
ISU1	GCCATCGCTTCTTCATCTGTTGC	TGGGAGAGAAAGATGCTTTGCG
