# Supplementary material for: TANDEM ZINC-FINGER/PLUS3 regulates phytochrome B abundance and signaling to fine-tune hypocotyl growth
Source: Plant Cell. 2022 Aug 5;34(11):4213–31. doi: 10.1093/plcell/koac236 (PMC9614508; doi:10.1093/plcell/koac236)
Supplement: koac236_Supplementary_Data [file Peer Review Report TPC2022RA00278DR1.pdf › koac236_Supplementary_Data/Peer Review Report TPC2022RA00278DR1.pdf]

TANDEM ZINC-FINGER-PLUS3 regulates phytochrome B Abundance and signaling to fine-tune hypocotyl growth

Weiwei Fang, Elisa Vellutini, Giorgio Perrella, and Eirini Kaiserli

Corresponding authors: *Eirini Kaiserli* *eirini.kaiserli@glasgow.ac.uk*

Review timeline:

**TPC2021-RA-00879**	Submission received:	Oct. 11, 2021
	1st Decision:	Nov. 22, 2022 *Decline with external review*
**TPC2022-RA-00278D**	Submission received:	Mar. 2, 2022
	1st Decision:	May 05, 2022 *Request revisions*
**TPC2022-RA-00278DR1**	Revision received:	Jun. 14, 2022
	1st Decision:	Jun. 14, 2022 *Accept*
	Final acceptance:	July. 28, 2022

REPORT: (The report shows the major requests for revision and author responses. Minor comments for revision and miscellaneous correspondence are not included. The original format may not be reflected in this compilation, but the reviewer comments and author responses are not edited, except to correct minor typographical or spelling errors that could be a source of ambiguity.)

**TPC2021-RA-00879**	**Submission received**	**Oct. 11, 2021**

**TPC2021-RA-00879**	**1st Editorial Decision – *Decline with external review***	**Nov. 22, 2021**

Thank you for choosing to send your manuscript entitled "TANDEM ZINC-FINGER/PLUS3 Fine-Tunes Hypocotyl Elongation in Red Light and Long Days by Regulating Phytochrome B and PIF4 Protein Abundance." for consideration at The Plant Cell. Your submission has been evaluated by members of the editorial board as well as expert reviewers in your field, and we regret to inform you that we are not able to recommend publication of this manuscript in its current form. We have not made this decision lightly. We have had input from multiple scientists, and have solicited post-review comments as well. Our present policy is to offer streamlined decisions and to not advise on the direction of the work by requesting extensive modifications or substantial additional experiments.

Your manuscript was assessed by three experts in the field. All found your work interesting. However, a common theme in the comments is that the reviewers would have liked more detail of the mechanisms by which phyB activity is affected by TZP and PCH1, and in particular, they did not find it sufficiently clear whether TZP and PCH1 only regulate phyB abundance or whether they also regulate phyB signaling. This is summarized by reviewer 3 in the comment that the manuscript "does not show how TZP and PCH1 work together to regulate red light sensing and hypocotyl elongation," and "it is not clear if TZP affects phyB signaling or stability, either via thermal reversion, or via an inhibition of degradation, both, or another way." The reviewers also make many other comments that would require attention, including more appropriate statistical analyses, and improved microscope images. Based on these comments, we are declining the current version of the manuscript. Nevertheless, we would be prepared to consider a revised version that takes account of all of the reviewers' comments, and includes new data that distinguishes effects on phyB signaling from effects on its stability. However, considering the amount of work involved and the extensive data set already shown, we would also understand if you decided to submit to another journal. If you decide to revise the story and resubmit to The Plant Cell, we will try to send it to the same reviewers.

TPC2022-RA-00278D Submission received Mar. 02, 2022

--- Reviewer comments (with **Authors responses in** blue):

We are grateful to the Reviewers for their very constructive comments.

Please find below a point-by-point response to every comment made by Reviewers 1, 2 and 3.

Reviewer #1 (Comments for the Author):

This is an interesting manuscript which shows that the transcriptional regulator TZP acts to stabilize phyB in nuclear photobodies and regulate PIF4 accumulation in red light and long days, thereby contributing to the regulation of hypocotyl elongation in these conditions. It builds on published findings showing a similar role for the TZP/phyB-binding protein PCH1. As such, this manuscript confirms the importance of the TZP/PCH1 complex in phyB signaling. The manuscript is generally well written and the majority of conclusions are supported by data. There are, however, a few conclusions which require additional evidence and a number of issues which would improve clarity for the reader.

1. Supplemental figure 2F does not convince the reader that the OXTZP/pch1 lines are expressing similar levels of TZP to OXTZP lines. Without this, hypocotyl phenotypes in OXTZP and OXTZP/pch1 lines cannot be compared, as these may result from different TZP levels. Furthermore, it is not clear that line 2-11 3 is even overexpressing TZP. Two bands are shown on the top western blot, but neither seem correspond to TZP. Later in the manuscript (Figure 4), line 1-12 is used as the authors claim that this has similar levels of TZP to the OXTZP control. TZP-GFP quantification should therefore be provided in Figure 4A and this line used for hypocotyl analyses comparing OXTZP to OXTZP/pch1 lines (eg. replace supplemental Figure 2A-D with data from line 1-12). Where pch1 is being compared to OXTZPpch1 (Figure 2), quantification of TZP levels in transgenic lines should be provided so the reader can see the extent of TZP expression compared to OXTZP.

> **RESPONSE: Thank you for your comment. We have added additional immunoblot analysis showing the TZP protein expression levels of all the lines used for hypocotyl assays and confocal imaging in this study (Supplemental Figure 2F-H). Semi-quantitative analysis of *OXTZP* and *pch1 OXTZP* can now be found in Supplemental Figure 2G-I).**

2. Although statistical analyses have been performed on hypocotyl data, only selected comparisons are shown on Figures. As such, the reader is unable to see the statistical significance of hypocotyl differences between other genotypes. For example, are pch1 hypocotyls significantly longer than Col-0 in SD (Figure 1B)? A lettering system which enables the reader to compare all genotypes simultaneously would be much more useful to the reader (eg. Hayes et al. 2019 Current Biology 29: 1669-1676).

> **RESPONSE: Thank you very much for your very helpful suggestion. We have now performed further statistical analysis and as suggested used a lettering system to represent significant differences by one-way ANOVA with Tukey's post hoc test among assessed samples (*P*<0.05) and updated all the graphs (hypocotyl elongation, protein quantification, transcript levels) throughout the manuscript.**

Additional minor issues to address:

Page 5. HFR1 is described as a growth promoting protein. HFR1 binds to and inhibits PIF activity.

> **RESPONSE: Please accept our apologies. We have revised all relevant sentences referring to HFR1 to "hypocotyl elongation marker genes".**

In Figure 1, what do 'A' and 'D' represent next to 'tzppch1' labels? This should be explained in the Figure legend.

> **RESPONSE: We apologise for this omission. We have now added the following text to Figure 1 caption : "*tzp pch1* A, D indicate two independent double mutant lines".**

There are a number of typos and omissions throughout the text (eg. line 107 should state 'and TZP'.

> **RESPONSE: We apologise for the typos. We have now proofread and corrected any errors.**

Line 205 mentions SD in supplemental Figure 3, but no SD data are provided. This should be added or the reference to SD removed.

> **RESPONSE: Thank you. We have revised the sentence.**

Figure 6D should be part of Figure 7, where it is referenced.

> **RESPONSE: Thank you for the insightful suggestion. We have now moved phyB localisation and quantification to Figure 7.**

Figure 7D. This model does not seem to correspond to the description in the figure legend or highlight the key findings of the manuscript. For example, it is not clear from the model that TZP binds to and stabilizes phyB, or that PIF4 promotes hypocotyl elongation. A clearer model should be provided, highlighting the key findings of the manuscript.

> **RESPONSE: Thank you. We have now revised the model (Figure 9).**

Supplemental Figure 3. Statistical analysis is mentioned in the legend but not shown on the graph. This should be added.

> **RESPONSE: We apologise for this omission. We have performed detailed statistical analysis on all graphs.**

Supplemental Figure 4. The legend states that asterisks represent significant differences between the indicated line and Col-0. This doesn't correspond with the description in the text (lines 323-326) and needs correcting.

> **RESPONSE: Thank you. We have now performed detailed statistical analysis on both hypocotyl and RT-qPCR graphs and used a lettering system to indicate significant differences between *tzp phyB*, Col-0 and *phyB*.**

The Methods section states that humidity and temperature were controlled for each experiment using soil-grown plants, but no details are provided. Temperature and humidity values should be added to either the methods or figure legends.

> **RESPONSE: We apologise for this omission. We have now added the values.**

Reviewer #2 (Comments for the Author):

In this manuscript, Fang et al. found that two previously known light signaling factors TZP and PCH1 interact with each other and synergistically modulate hypocotyl growth in long day photoperiodic conditions as well as in red light. They showed that TZP and PCH1 regulate PIF4 protein abundance, underlying the functional relationship between them. Moreover, PCH1 regulates the sub-nuclear localization pattern of TZP under red light. Furthermore, they showed that TZP promotes light-dependent phyB protein stability to positively regulate phyB signaling. Overall, the manuscript is well written in a clear manner. However, some of the data are of low quality and the underlying new mechanism is lacking. Below are some concerns/suggestions for improvement of the manuscript:

Major comments:

TZP has been reported to colocalize and interact with phyB (Kaiserli et al., 2015). PCH1 binds phyB in a red light-dependent manner, and co-localizes with phyB into photobodies. PCH1 also alters PIF4 levels and regulates light-responsive gene expression and photoperiodic response (Huang et al., 2016). This study mainly provides physiological phenotypes (tzp pch1 double mutant and other genetic interactions; Figure 1, 2 and 5), PIF4 protein level in the tzp pch1mutant (Figure 3), TZP nuclear body phenotype in the pch1 mutant (Figure 4), phyB protein levels in tzp and OXTZP (Figure 6 and 7). However, the major weakness of this story is the lack of new mechanism or mainly based on the known mechanism.

> **RESPONSE: We apologise for not emphasizing the novel findings of this study. In addition to uncovering an additive action between TZP and PCH1, we show that PCH1 is important for TZP nuclear body abundance and TZP function. Furthermore, we show that TZP is a regulator of phyB protein abundance and phyB nuclear body morphology and function. As a result, TZP modulates not only phyB but also PIF4 protein abundance and therefore leads to fine-tuning hypocotyl growth regulation in response to red light and long days.**

Figure 1A-C. The author state that "the loss of function of both PCH1 and TZP led to a novel elongated hypocotyl phenotype that has not been previously reported for the single tzp nor pch1 mutants (Line136)." The "novel" elongated hypocotyl phenotype may be largely due to the light intensity the author used. Therefore, the hypocotyl length should be determined in a series of light intensities.

> **RESPONSE: Thank you for this observation, We have now added data showing hypocotyl elongation assays at the light conditions previously used to describe the *pch1* single mutant (Huang et al., 2016) (LD white light 80 µmol m-2 s-1) in addition to LD white light of 60 µmol m^{-2} s^{-1}). Our new data shown in Supplemental Figure 1E and H are consistent with the original observation that *tzp pch1* has elongated hypocotyls compared to the respective single mutants that resemble Col-0.**

Figure 1D, ATHB2 expression levels were reduced in the tzp and pch1 single mutants compared to Col wild-type, how could their transcript levels increase in the tzp pch1 double mutant?

As the authors generated OXTZP transgenic lines in this study, multiple lines should be provide, in addition to in the pch1 background, to show the consistence of their expression and phenotypes.

> **RESPONSE: Thank you for your comment. It is possible that the loss of function of both TZP and PCH1 is required for causing an upregulation of ATHB2.**

Multiple independent *OXTZP* lines have been previously reported in Loudet et al., 2008, Kaiserli et al., 2015 and Perrella et al., 2018, therefore we used a representative well-characterised homozygous line for all our assays.

Figure 3D. PIF4 showed specific expression pattern at both transcription and protein level. Whether these data accord with previous results? More importantly, the protein level of PIF4 should be quantified to attain an exact conclusion.

RESPONSE: Thank you for your comment. Yes, our data agree with previous reports and have added the relevant references regarding *PIF4* transcript and PIF4 protein regulation (Nozue et al., 2007, Nomoto et al., 2013). We have now added semi-quantitative analysis of PIF4 protein levels in Supplemental Figure 3B-C.

Figure 4C, D. The hypocotyl phenotype was examined under the red light intensity of 1 μmol m-2 s-1. This means *TZPGFP pch1* and *TZP-GFP* exhibited strong phenotype under this condition. However, confocal image analysis of nuclear bodies per nucleus were performed under the red light intensity of 20 μmol m-2 s-1. The author should conduct confocal image analysis under 1 μmol m-2 s-1 red light intensity to reinform the conclusion.

RESPONSE: Thank you very much for the suggestion. We have performed confocal image analysis of TZP versus TZP pch1 at 1 μmol m-2 s-1 and added the relevant images and quantitative data analysis in Figure 4A-B.

Furthermore, we have also added hypocotyl elongation analysis at 20 μmol m-2 s-1 and 40 μmol m-2 s-1 (in addition to 1 μmol m-2 s-1). These data are shown in Suppl. Figure 1C-D and H and are consistent with our original observations that *tzp pch1* has elongated hypocotyls compared to the respective single mutants that resemble Col-0.

Figure 5 and 6. TZP showed no significant regulation of hypocotyl growth under red light in a wild type background. However, phyB protein levels are higher in tzp compared to WT. Given that the critical role of phyB in photomorphogenesis. The data is really puzzling. The genetic interaction between PHYB and TZP may be masked by the ecotypic effect of 35S driving PHYB or TZP. Therefore, it would be interesting to depict their genetic relationship using the phyB tzp double mutant. phyB protein levels were relatively equal (or slightly reduced) in the tzp mutant under red light (Figure 6A), whereas phyB protein as almost undetectable in tzp compared to Col under white light (Figure 7A). Are these really the true cases? Please explain.

In Figure 6C, phyB mRNA level is significantly reduced in the tzp mutant. Then how the conclusion "TZP controls phyB protein but not mRNA abundance" was made?

RESPONSE: We completely agree that it is puzzling how neither *tzp* nor *OXTZP* lead to elongated hypocotyl phenotypes in red light, even though phyB levels are decreased or increased respectively. Examining the *tzp phyB* mutant showed red light mediated hypocotyl elongation responses similar to *phyB* (Figure 5A). These findings indicate that *phyB* is epistatic to *tzp* in response to red light, which is in accordance with the data published previously (Zhang et al., 2018). Supplemental Figure 6 (A, E, F, G) shows semi-quantitative analysis of immunoblot assays monitoring phyB protein. Consistently, *tzp* mutants lead to a lower abundance of phyB in red, darkness, and white light and *OXTZP* leads to higher phyB levels compared to Col-0.

We have provided more biological replicates, detailed statistical analysis and additional data to study *PHYB* mRNA in *tzp* (Supplmental Figure 6D-E). Our data shows that *PHYB* mRNA abundance is not significantly changed in *tzp*.

The working model in Figure 7D is too simple to understand. It is hard to see the fine-tune actions by TZP and PCH1. Also, please include the SD/LD and Red light conditions.

RESPONSE: Thank you. We have now revised the model (Figure 9).

Minor points:

A recent paper on TZP-HY5 regulation published in Plant Cell (Li et al., 2021) should be properly cited.

RESPONSE: Cited.

Figure 1 D-E. The data should be shown as means {plus minus} SD of three biological replicates. Other qPCR quantification, including Figure 5C, should be determined the same. Please indicate whether biological or technical repeats for RT-qPCR results.

RESPONSE: We have performed further statistical analysis and used a lettering system. All graphs and related figure captions have been revised and also indicate the type of repeats.

In Figure 2, I could not find TZPGFP or OXTZP-GFP as indicated in the figure legend.

RESPONSE: Apologies. Corrected.

Figure S2F, the quality of WB (especially in the genetic crossing lines) is not good. Please provide a better one. Also add MW in the gel.

RESPONSE: Please accept our apologies. We have added additional immunoblot analysis showing the TZP protein expression levels of all the lines used for hypocotyl assays and confocal imaging in this study (Supplemental Figure 2 F-G). Semi-quantitative analysis of *OXTZP* and *pch1 OXTZP* can now be found in Supplemental Figure 2 G-I).

Add MW to related WB in figures 3, 4, 6, and 7.

RESPONSE: MW markers have been added on all immunoblot images.

Figure 3D, what is RbsC?

RESPONSE: Corrected to RbcS.

Figure S3C PIF4 transcription patterns could be moved to Figure 3, so that the mRNA and protein levels could be easily compared.

RESPONSE: Done. Now in Figure 3F.

Figure 4A, 6C, 7A, the loading controls by RbcS staining are of low quality, WB with anti-UGP is suggested. And quantify the GFP bands. The two graphs in Figure 4C and Figure 4D could be combined, respectively.

RESPONSE: All immunoblots loading controls requested have been replaced with UGP.
Figure 4A has also been repeated and multiple immunoblots and quantification are shown in Suppl.
Figure 4F-I.

Figures 4C and D have been moved to the supplement (Supplemental Figure 4A-B) and high-resolution images and quantification is shown in Figure 4.

The WB of anti-phyB in Figure 6A is redundant with that in Figure 3A?

> **RESPONSE: That's correct but we would like to retain both for a direct comparison to the PIF4 western blot (Fig. 6B) performed under the same conditions.**

107 TZP is missing?

> **RESPONSE: Apologies. Revised to: "PCH1 inhibits the thermal reversion of phyB and acts as a structural component for phyB nuclear body formation".**

Change the double mutant "*tzppch1*" to "*tzp pch1*", same issue to other double transgenic/mutant lines.

> **RESPONSE: Changed.**

120-121 "......the genetic relationship between these two proteins" is not correct.

> **RESPONSE: Apologies. Revised to : "we investigated the genetic relationship between these two components".**

141 "Hypocotyl assays" is not suitable.

> **RESPONSE: Apologies. Revised to : "Hypocotyl elongation assays"**

198, 367 "....... phyB protein level ......" is suggested.

> **RESPONSE: Thank you. Revised.**

The title could be modified.

> **RESPONSE: We have modified the title to "TANDEM ZINC-FINGER-PLUS3 Regulates Phytochrome B Abundance and Signaling to Fine-tune Hypocotyl Growth"**

Reviewer #3 (Comments for the Author):

The authors present an interesting work where they show a double mutant tzp pch1 with a genetic interaction. Both proteins are known to regulate the activity of phytochromes and both proteins are found in subnuclear phytochrome photobodies. The authors also show varying evidence that connects TZP function to the regulation of phyB and PIF4. However, it is not made clear if this is due to the direct stabilization of phyB or due to the regulation of phyB activity, or both. This means that the authors show no clear way how the genetic interaction of pch1 and tzp leads to the phenotype presented in fig. 1.

I think that the simplicity of the model in figure 7D is indicative for the lack of deeper understanding that this study offers.

This study would be greatly enhanced if the authors could show more how TZP and PCH1 affect phyB function, and specifically if TZP affects the activity and signaling of phyB, as opposed to just the protein amounts.

RESPONSE: Thank you for your constructive comments. We apologise for not emphasizing and clarifying the novelty of our findings of this study. In addition to uncovering an additive action between TZP and PCH1 through genetic interactions, we show that PCH1 is important for TZP nuclear body abundance and TZP function. Furthermore, we show that TZP is a regulator of phyB protein abundance and phyB nuclear body morphology and signaling. As a result, TZP modulates not only phyB but also PIF4 protein abundance and therefore leads to fine-tuning hypocotyl growth regulation in response to red light and long days.

To further understand how TZP regulates phyB abundance and/or signaling, in addition to all the comments suggested by Reviewers 1, 2 and 3, we extended this study, as suggested, by performing detailed confocal image analysis on TZP, phyB as well as the constitutively active phyB^{Y276H} (YHB). Our new data indicate that TZP positively regulates the protein abundance of WT phyB and to a much lesser extent of YHB (Supplemental Figure 8). However, the effect of TZP on phyB nuclear body morphology and abundance is retained in both *phyB* and *YHB*, indicating that TZP also plays a role in stabilising phyB photobodies in a similar manner to HEMERA and therefore leading to the observed effects on PIF4 abundance. This supports the hypothesis that TZP affects phyB signaling leading to changes in phyB and PIF4 abundance.

We have now revised the model (Figure 9).

First of all, increased phyB protein amounts do not stroke with increased PIF4, which makes it more likely that phyB activity is modulated. The sharp increase in PIF4 at end of night hint towards a faster thermal/dark reversion of phyB. And the nuclear body analysis of Figure 4D suggests that a similar thing is happening with the TZP bodies.

The authors state in line 302: "under conditions of excess phyB the loss of TZP may hinder phyB function in regulating hypocotyl growth inhibition." This would suggest a role of TZP in modulating phyB activity. A mechanism through which phyB protein amounts and activity regulation might be reconciled could be via the mutual PIF phyB degradation after PIF phosphorylation, as described by Ni et al. 2014 (Science). I think the authors should try to address this possibility more.

There are some more contradictions within the argumentation:

Line 467 "Our study proved that TZP can protect phyB from being degraded, however, the mechanism by which TZP exerts this function remains unclear."

Line 433 "Our results showed that tzppch1 led to elevated PIF4 protein levels throughout a long day photoperiod, which suggests that even at daytime (ZT0-16) when phyB is abundant, its effect on degrading PIF4 is largely attenuated in the absence of TZP and PCH1 (Figure 3D)."

This suggests that TZP and PCH1 would play a negative role in regulating phyB activity, even though TZP promotes phyB protein stability and promotes nuclear body formation (of phyB).

This is a problem of this study. There are results that show that TZP modulates the amount of phyB present, however, they do not seem to correlate with the amount of PIF4 present in the *tzp pch1* double, which is also increased. Therefore, it's much more likely that activity of phyB is modulated. However this is not properly addressed in this study

Figure 6D is very important in this argumentation, since this is the only result where we could possibly correlate phyB protein amounts with phyB activity.

Line 388: "The subnuclear localization pattern of phyB was also impaired in *tzp OXPB*. More specifically, *tzp OXPB* exhibited a reduced number of photobodies compared to *OXPB* (Figure 6D)."

This is not what the authors quantified. They show a log scale of the nuclear body area. I don't think this is a fair comparison if the intensities of the PHYB-CFP are so different between these two lines. With the microscopy used, it is not possible to spatially resolve such photobody size differences, especially when increased intensity artificially increases the perceived size of these nuclear bodies. Also, photobodies are still visible in the right panel of this image, although they appear much weaker. Firstly, the authors should actually count the number of photobodies. Secondly, they should give more information on how imaging was performed (which cells, which objective) and how the quantification was done. Thirdly, why stop here? Why not cross the *tzp pch1* mutant with this PHYB-CFP line.

> **RESPONSE: Thank you for your very insightful points and suggestions.**
>
> **We have performed more detailed imaging and analysis of phyB nuclear bodies in WT and *tzp* mutants. Higher resolution image, quantification of the number of nuclear bodies per nucleus and statistical analysis showed that phyB nuclear body abundance is impaired in *tzp* in response to two different red light intensities (Figure 7A-D).**

The authors themselves propose an excellent experiment through which the problem of phyB stability vs degradation could be resolved: line 392: "One way to further test this is to compare the photobody formation of the constitutively active phyBY276H (Su and Lagarias, 2007)."

I think the authors hit the nail right on the head here.

> **RESPONSE: We examined the nuclear body formation and protein abundance of the constitutively active phyB^{Y276H} in the presence and absence of functional TZP. The data shown in Figure 8 (A-F) and Supplemental Figure 8 (A-B) suggest that TZP affects the number and size of phyB^{Y276H} whereas it has a minor effect on phyB^{Y276H} protein levels. Although phyB^{Y276H} can form photobodies in *tzp*, their size is reduced and their number increased (similar to the phenotype reported for *hmr* (Chen et al., 2010). Therefore, TZP potentially affects the stability of phyB photobodies regardless of the total protein abundance. PCH1 is reported to act as a structural component for phyB photobodies and phyB^{Y276H} cannot form photobodies in *pch1*. Perhaps TZP has an accessory role in PCH1-mediated phyB nuclear body formation or a similar role to HMR that promotes phyB photobody formation and PIF degradation.**

Furthermore, there is the issue with transcriptional control of PIF4 and phyB by TZP:

Figure 5C suggests that transcript levels of PIF4 are modulated by TZP, while in supp figure 3C the authors say that's not the case. However, if you look at ZT4 in this figure, you would get the same effect as in Fig. 5C. Even within *PHYBox*, *PIF4* expression is still upregulated in *tzp*. therefore, it could be that the effect of PIF4 stability during the day and night is regulated transcriptionally via TZP, not directly related to phyB.

RESPONSE: We cannot exclude the possibility that TZP also regulates *PIF4* at the transcriptional level but to a lower extent. Therefore, we also added this in the text.

Then about *PHYB* expression:

Line 356: "..while *tzp* showed slightly lower *PHYB* expression (Figure 6E)." This result seems to show that *PHYB* expression in *tzp* is half that of wild type.

RESPONSE: Thank you for your comment.

We have provided more biological repeats, detailed statistical analysis and additional data to study *PHYB* mRNA in *tzp* (Supplemental Figure 6D-E). Our data shows that *PHYB* mRNA abundance is not significantly changed in *tzp.*

As in figure 1 D,E, the authors are using a wild type control. I think this is a good reason to use the ΔΔCT method of calculating relative expression, as to normalize against the wild type control as well, and not only against the housekeeping gene.

RESPONSE: Thank you for the suggestion. We have revised the RT-qPCR data and used the ΔΔCT method for calculating relative expression. We have performed further statistical analysis and used a lettering system. All graphs and related figure captions have been revised and also indicate the type of repeats.

Line 358: "..and if any, PHYB transcript was even slightly higher in tzpOXPB than its parental line (Figure 6F). Thus, the possibility that a transcriptional mechanism may underlie the observed variations in phyB protein levels is negligible."

I think this is an incorrect interpretation. In tzp transcription of phyB is down, while in TZPox phyB transcription is up. This would argue for transcriptional control of phyB by TZP. That would correspond well with the reduced protein amounts as well. Possibly this could occur via regulation of phyB activity, possibly modulating its own transcription via downstream targets.

RESPONSE: Thank you. This is a possibility. The sentence has been revised.

To summarize, the two biggest shortcomings of this study are: It does not show how TZP and PCH1 work together to regulate red light sensing and hypocotyl elongation.

It is not clear if TZP affects phyB signaling or stability, either via thermal reversion, or via an inhibition of degradation, both, or another way.

I think that good ways to address this problem are to increase the amount and quality of the microscopy analysis.

Right now, it is unclear how temporally and spatially, tzp and TZPox affect phyB nuclear body formation.

Especially figure 6D is of rather poor quality at the moment.

It would be great if we know what the effect of tzp is on phyB activity during the day! For example, extend the analysis of 4C and D to the tzp phyB-CFP line.

Think of doing the MG132 treatment in combination with PHYB-CFP microscopy. Then it will be clear how activity and degradation might correlate.

RESPONSE: In addition to performing higher resolution image, quantification of the number of nuclear bodies per nucleus and statistical analysis on phyB nuclear bodies in WT and *tzp* mutants (Figure 7A-D), we also examined the effect to MG132 treatment (identical to the one used for immunoblot analysis of phyB) on phyB nuclear bodies in the absence and presence of TZP (Supplemental Figure 7). MG132-treated seedlings showed a decrease in phyB photobodies in *tzp*, however, statistical analysis determined that this difference was not significant. Therefore, these results need to be interpreted with caution as the effect of MG132 in photobody formation is not fully established. In addition to its role in inhibition of proteasomal function, MG132 may also affect phyB nuclear body formation by an unknown mechanism.

Additionally, it is interesting how *tzp pch1* would affect that. Presumably we would see very little nuclear bodies in this double mutant, going from what is known for *pch1.*

RESPONSE: We would love to do this experiment, however, generating homozygous *tzp pch1 OXPB* lines would require at least 6 additional months and therefore could delay the publication of our findings. In addition, we believe that these lines would not necessarily provide us with further insight on the mechanism of TZP action as we already know that phyB nuclear bodies are hugely impaired in *pch1*.

Technical remarks:

Improve the statistics. Since the authors perform one-way ANOVA, it is possible to post-hoc calculate the significance between individual averages, and then to show these differences with letter-based categories, instead of asterisks.

RESPONSE: Thank you very much for your very helpful suggestion. We have now performed further statistical analysis and as suggested used a lettering system to represent significant differences by one-way ANOVA with Tukey's post hoc test among assessed samples (P<0.05) and updated all the graphs (hypocotyl elongation, protein quantification, transcript levels) throughout the manuscript.

use Δ-ΔCT method for calculating relative expression.

RESPONSE: Thank you very much for your suggestion. We have now reanalysed the gene expression data by using the Δ-ΔCT method.

Describe in a more detailed manner how microscopy was performed and which cells were imaged, how quantification was done. Also the image quality can be improved, show higher resolution images (pixels) please.

RESPONSE: Thank you for your comment. We have now provided more information on how imaging was performed and increased the resolution by using higher magnification (Confocal Imaging and analysis section and in all relevant figure captions).

Minor remarks:

line 77: "directly interacts with phytochromes"

RESPONSE: Corrected.

Figure 1A,B: the LD SD hypocotyl experiments are a bit ambiguous with respect to the *pch1* mutant. Previously it was shown that *pch1* has a longer hypocotyl in SD (Huang 2016). In this experiment, the error bar is quite large and subsequently there is no statistically significant effect. Therefore this experiment has to be repeated. If possible with a bigger n than 15.

RESPONSE: We have performed statistical analysis and *pch1* is significantly longer than Col-0, however, if needed we can provide an alternative graph.

line 107: "Since mass spectrometry analysis revealed that PCH1, phyB and TZP co-precipitate together (Huang et al., 2016b)"

line 122 and figure 1a,b: Can the authors explain what the difference is between pch1tzp D and A?

RESPONSE: We apologise for this omission. We have now added the following text to Figure 1 legend: "*tzp pch1* A, D indicate two independent double mutant lines".

Figure 1C: It is not clear to me which groups have been compared in post-hoc ANOVA analysis due to the placement of the horizontal lines and asterisks. Also, what kind of post-hoc test has been used?

Figure 1C: The authors should have compared all averages with all (with a post-hoc Tukey for example) and ascertain whether *pch1* is different from *pch1 tzp* double.

Figure 1D,E: Since the authors have Col-0 wild type as a control I think it is better is the Δ-ΔCT method for calculating relative expression is used.

RESPONSE: Thank you very much for your very helpful suggestion. We have now performed further statistical analysis and as suggested used a lettering system to represent significant differences by one-way ANOVA with Tukey's post hoc test among assessed samples (*P*<0.05) and updated all the graphs (hypocotyl elongation, protein quantification, transcript levels) throughout the manuscript.

Figure 2A, line 176: "*OXTZP pch1* lines showed a reversal of the *pch1* phenotype indicating that *TZP* overexpression can partially rescue the elongated hypocotyl phenotype of *pch1* in red light (Figure 2B)" I think this is a partial reversal. it seems more as though *OXTZP* is epistatic to *pch1.*

RESPONSE: Thank you for your comment. We have now revised the sentence: "*pch1 OXTZP* lines showed a partial reversal of the *pch1* phenotype indicating that *TZP* overexpression can rescue the elongated hypocotyl phenotype of *pch1* in red light or OXTZP is potentially epistatic to PCH1".

Figure 3A: I would appreciate it if the authors could show a little bit more of this blot, since the signal of the phyB bands is partially cut off and obscured due to the image cropping. The smear is also part of the signal!

RESPONSE: As requested, the uncropped blot as Supplemental Figure 3A.

line 195: "Yet, this TZP-dependent phyB accumulation is impaired in *OXTZP pch1* (Figure 3A)"

Based on this one blot, I think it is too strong a conclusion. The difference appears to be very small, and also part of the blot is obscured making it more difficult to ascertain. It would help, if the authors would show the repeat experiments and full blots in the supplemental.

RESPONSE: Thank you for your comment. We have now included semi-quantitative measurements of the immunoblot repeats (Figure 3B) to solidify this conclusion.

Figure 4: Which cells have been imaged? They all seem to be at different scales and therefore could be different cell types. In the top-left panel it seems as though this cell has a multitude of smaller bodies. Is this a phenotype that is consistent? Can the authors specify based on what they quantified a body? If the plants were live-imaged, would that not affect the maintenance of nuclear bodies, since the microscope environment will be dark.

RESPONSE: We have revised Figure 4 with higher resolution images and quantification. Detailed description of how the imaging was performed has been added in the figure caption and methods section.

line 281: What does the reference of Perrella 2021 add in this remark? This is a methods section from a book.

RESPONSE: Apologies. The reference has been removed.

Line 384, Figure 7C: "On the contrary, dark-grown seedlings showed unaltered phyB protein accumulation with or without MG132."

I believe it appears relatively clear that MG132 treatment increases *OXPHYB* levels in Col-0 background in the dark. In the *tzp* mutant this effect is gone. This would argue for a role of *tzp* in the thermal/dark reversion of phyB

RESPONSE: You are absolutely right. We apologise for the misinterpretation. We have now revised this statement:

"On the contrary, *tzp* dark-grown seedlings showed unaltered phyB protein accumulation with or without MG132."

Line 469: This is highly speculative and there is no clear evidence for this. Maybe tone down the "highly likely"?

RESPONSE: Apologies. Revised.

TPC2022-RA-00278DR1	1st Editorial decision – *Request revisions*	May 05, 2022

We have received reviews of your manuscript entitled "TANDEM ZINC-FINGER/PLUS3 Regulates Phytochrome B Abundance and Signaling to Fine-tune Hypocotyl Growth." Thank you for submitting your best work to The Plant Cell. The editorial board agrees that the work you describe is substantive, falls within the scope of the journal, and may become acceptable for publication, pending revision and potential re-review.

We ask you to pay attention to the following points in preparing your revision:

The reviewers, particularly reviewer 2, found the revised version of the manuscript improved and the effects shown on PhyB and PIF4 protein abundance of interest. However, both reviewers emphasize that there is still a lack of mechanistic understanding of how TZP and PCH1 work together. We agreed in the post-review consultation not to request more data on this point, but to ask that the text be revised to make this clearer in the abstract and the discussion. We also still found

the model in figure 9 unclear, probably because of the mechanistic uncertainty. We ask that you revise this again or remove it. In addition, edit the text to take account of all other comments of the reviewers, but we are not asking for additional data at this stage

TPC2022-RA-00278DR1	1st Revision received	Jun. 14, 2022

Response to Reviewer comments

We are extremely grateful to all reviewers for the insightful comments and suggestions that we believe have greatly improved our manuscript.

Reviewer #2 (Comments for the Author):

Although the authors performed some additional experiments and added explanations, they still did not address my two major concerns in the previous review comments.

1) However, the major weakness of this story is the lack of new mechanism or mainly based on the known mechanism. Authors' response: As a result, TZP modulates not only phyB but also PIF4 protein abundance and therefore leads to fine-tuning hypocotyl growth regulation in response to red light and long days.

My comment: In my opinion, this issue is important for this paper. Actually, TZP has been reported to colocalize and interact with phyB (Kaiserli et al., 2015). PCH1 binds phyB in a red light-dependent manner, and co-localizes with phyB into photobodies. PCH1 also alters PIF4 levels and regulates light-responsive gene expression and photoperiodic response (Huang et al., 2016).

RESPONSE: We really appreciate the comments of Reviewer 2 to help us improve the manuscript. Please allow us to clarify and highlight how this study is significantly novel and expands our knowledge in the current understanding of TZP, PCH1 and phyB regulation:

- **Kaiserli et al., 2015 showed that "phyB is required for TZP nuclear body formation and function in photoperiodic flowering control".**

  The role of TZP in regulating phyB has not been reported before. This study shows that TZP is a positive regulator of phyB protein abundance, nuclear body formation and signaling. Furthermore, we report a new role for TZP in red light mediated hypocotyl elongation.

- **Huang et al., 2016 showed that "PCH1 co-localizes with phyB into photobodies and affects PIF4 levels" specifically under short day photoperiodic conditions. We have revealed a new function for PCH1 in long-days. We also show that the absence of**

both TZP and PCH1 leads to an increase in PIF4 protein levels in long days and red light and therefore unmasks a novel role for TZP in red light mediated regulation of hypocotyl elongation and phyB-mediated signaling.

We have highlighted the novelty of our findings in the abstract, introduction, results and discussion sections.

2) Figure 1D, *ATHB2* expression levels were reduced in the *tzp* and *pch1* single mutants compared to Col-0 wild type, how could their transcript levels increase in the *tzp pch1* double mutant?

Authors' response: It is possible that the loss of function of both TZP and PCH1 is required for causing an upregulation of ATHB2.

My comment: It's still unknown or well explained.

RESPONSE: To further solidify our results regarding *ATHB2* expression, we have included additional repeats presented in Figure 1D. *ATHB2* transcript levels are lower in *tzp* and *pch1* single mutants compared to *tzppch1* double mutant, but comparable to Col-0. *ATHB2* is known to be a target locus for multiple light (shade, blue, red light), hormone (auxin) and clock pathways regulating hypocotyl growth, therefore the *ATHB2* expression pattern observed in the single *tzp* or *pch1* mutants may be masked or compensated by other factors.

We have added this clarification in lines 170-174.

Reviewer #3 (Comments for the Author):

Comments:

The resubmitted manuscript has addressed a lot of questions and the data has improved in critical places. This manuscript contains a lot of interesting, important data and raises many questions. The authors show clearly that tzp enhances the effect of pch1. Furthermore, TZP overexpression is able to reduce the pch1 hypocotyl phenotype.

It is great to see how PIF4 abundance changes during the day and how the pch1 tzp mutant has a much higher PIF4 abundance during the night.

We are grateful to Reviewer 3 for the very positive comments.

tzp has a strong effect on phyB abundance, which is a great result. But what is extremely puzzling is that *tzp* itself has no discernible phenotype in red light. Maybe phenotypes will only become apparent in different conditions?

RESPONSE: This has indeed puzzled us and other labs working on TZP for years. We have examined the phenotype of *tzp* mutants at 1, 20 and 40 µmol m^{-2} s^{-1} red light and

showed no statistically significant differences compared to WT even though *tzp* leads to a decrease in phyB protein abundance. Whether other phytochromes or downstream signaling components compensate for the decrease in phyB protein in *tzp* is a possible explanation that could be investigated in future studies.

This is now highlighted in the discussion (lines 589-594).

The finding that nuclear bodies of YHB are smaller, but not absent in *tzp*, shows that *tzp* most likely does have an effect on nuclear body formation of phyB, in addition to the effect on phyB degradation. Whether that is directly linked is unclear, but possible, something the authors acknowledge.

At the beginning of the discussion, the authors state the following: "Firstly, our data uncover a novel inter-dependent role for TZP and PCH1 in controlling hypocotyl elongation in long days as well as in response to red light.."

This inter-dependent role is very unclear. I think the authors have addressed quite well some of the effects of the *tzp* mutation on phyB nuclear bodies, and they show the effects of the *tzp pch1* double mutation on PIF4 abundance, but it is unclear how TZP aids PCH1. Indeed the authors show that *pch1* affects TZP nuclear bodies, but how do they cooperate? There is no clear mechanism described how these two proteins cooperate. Now I acknowledge that that is a study in itself, however I think it should be made clear in the abstract and discussion that this is completely unknown and simply guesswork.

Where the model presented in the previous manuscript was very simplistic, the model presented now is in Figure 9 is somewhat vague. TZP simply promotes phyB? Is that it? Looking at the data of PIF4 abundance it seems that PCH1 and TZP cause a lot of PIF4 to be retained in the night, something that is hardly reflected in the model. Do TZP and PCH1 not have a role in the dark in stabilizing phyB? And what about the finding that PCH1 regulates TZP nuclear bodies? This is missing in the model.

RESPONSE: We appreciate that the relationship between TZP and PCH1 is complex and difficult to depict in a model at this stage. We have no evidence of a direct interaction between TZP and PCH1 and it is likely that their synergistic action in controlling phyB-dependent hypocotyl growth in red light and long days is mediated through intermediate factors including phyB and PIFs.

We have revised the abstract (Lines 25-34), introduction (lines 108-112) and discussion (lines 471-475; 618-622) to clarify this point and removed the model as also suggested by the Editor as it is premature at this stage.

Strengths: interesting data, new findings on TZP as a modulator pf phyB stability and nuclear body formation. The profound effect of pch1 tzp on PIF4 abundance in the night. Weakness: The lack of understanding how PCH1 and TZP work together

Minor comments:

Figure 5A, statistical analysis missing?

RESPONSE: Thank you for pointing out this omission. We have now added the statistical analysis.

Figure 6D: labelling error: In the light, OXTZP is switched with tzp

RESPONSE: Thank you for noting this error. We have corrected the labelling.

All changes, including the ones mentioned above, are highlighted in the main text and additional information has been added regarding the materials, methods, statistical analysis and replicates according to the author revision checklist. Furthermore, Supplemental Figure 9 has been incorporated in the main manuscript as Figure 9 based on the fact that the data support one of the major claims rather than supporting a figure.

**TPC2022-RA-00278DR1**	**1st Editorial decision –** ***Request revisions***	**May 05, 2022**

We are pleased to inform you that your paper entitled "TANDEM ZINC-FINGER-PLUS3 Regulates Phytochrome B Abundance and Signaling to Fine-tune Hypocotyl Growth" has been accepted for publication in The Plant Cell, pending a final minor editorial review by journal staff. At this stage, your manuscript will be evaluated by a Science Editor with respect to its presentation of scientific content, compliance with journal policies, and presentation for a broad readership.

**Final acceptance from Science Editor**	**Jul. 28, 2022**
